# Computational Study of SARS-CoV-2 RNA Dependent RNA Polymerase Allosteric Site Inhibition

**DOI:** 10.3390/molecules27010223

**Published:** 2021-12-30

**Authors:** Shah Faisal, Syed Lal Badshah, Bibi Kubra, Mohamed Sharaf, Abdul-Hamid Emwas, Mariusz Jaremko, Mohnad Abdalla

**Affiliations:** 1 Department of Chemistry, Islamia College University Peshawar, Peshawar 25120, Pakistan; faisalvbs@gmail.com (S.F.); kubrakha621@gmail.com (B.K.); 2 Department of Biochemistry and Molecular Biology, College of Marine Life Sciences, Ocean University of China, Qingdao 266003, China; mohamedkamel@azhar.edu.eg; 3 Department of Biochemistry, Faculty of Agriculture, AL-Azhar University, Nasr City, Cairo 11751, Egypt; 4 Core Labs, King Abdullah University of Science and Technology (KAUST), Thuwal 23955-6900, Saudi Arabia; abdelhamid.emwas@kaust.edu.sa; 5 Smart-Health Initiative (SHI) and Red Sea Research Center (RSRC), Division of Biological and Environmental Sciences and Engineering (BESE), King Abdullah University of Science and Technology (KAUST), Thuwal 23955-6900, Saudi Arabia; 6 Key Laboratory of Chemical Biology (Ministry of Education), Department of Pharmaceutics, School of Pharmaceutical Sciences, Cheeloo College of Medicine, Shandong University, 44 Cultural West Road, Jinan 250012, China

**Keywords:** SARS-CoV-2, allosteric site, RNA dependent RNA polymerase, naringoside, myricetin

## Abstract

The COVID-19 pandemic has caused millions of fatalities since 2019. Despite the availability of vaccines for this disease, new strains are causing rapid ailment and are a continuous threat to vaccine efficacy. Here, molecular docking and simulations identify strong inhibitors of the allosteric site of the SARS-CoV-2 virus RNA dependent RNA polymerase (RdRp). More than one hundred different flavonoids were docked with the SARS-CoV-2 RdRp allosteric site through computational screening. The three top hits were Naringoside, Myricetin and Aureusidin 4,6-diglucoside. Simulation analyses confirmed that they are in constant contact during the simulation time course and have strong association with the enzyme’s allosteric site. Absorption, distribution, metabolism, excretion and toxicity (ADMET) data provided medicinal information of these top three hits. They had good human intestinal absorption (HIA) concentrations and were non-toxic. Due to high mutation rates in the active sites of the viral enzyme, these new allosteric site inhibitors offer opportunities to drug SARS-CoV-2 RdRp. These results provide new information for the design of novel allosteric inhibitors against SARS-CoV-2 RdRp.

## 1. Introduction

Coronavirus disease (COVID-19) appeared at the end of 2019 in Wuhan China [1], and is caused by the virus SARS-CoV-2. COVID-19 became a pandemic, causing millions of deaths [2,3,4]. The ability of β-coronaviruses (CoVs) to potentially cause a pandemic was apparent prior to 2019 [5]. In 2002, an atypical pneumonia-like viral disease spread among people in China; it was called severe acute respiratory syndrome (SARS) [6]. Then, in April 2012, a new CoV, called MERS-CoV, was reported to have infected people in the Middle East region [7], triggering severe acute respiratory syndrome in the infected patients [8]. SARS-CoV-2 is therefore a new CoV family member [9].

Most of the CoVs are transmitted to humans from zoonotic sources [10], and the same is the case with SARS-CoV-2. It is suspected to have been transmitted to humans from animals, such as bats [11]. Unlike the previous CoVs, which have infected thousands of people and have low transmissibility from person to person, SARS-CoV-2 has a high rate of transmission due to better interaction of its binding domain with host receptor cells [12]. This result in a higher rate of viral transmission of SARS-CoV-2 and millions of infections and deaths have been recorded across the globe.

SARS-CoV-2 is an RNA virus; compared to other RNA-based viruses, its genome is relatively larger in size. Among all RNA virus families, CoVs have the largest genomes (26–32 kb) [13]. A multi-subunit replication/transcription machinery is used by CoVs to make its genomic copies and pack it into virus particles [13]. Cellular entry of SARS-CoV-2 is mediated by the interaction of the virus S-protein and the host hACE-2 receptor [14,15]. After transfer into the host cell, translation of the viral genome produces several proteins, known as non-structural proteins (NSP). NSP translation occurs from the open reading frames (ORFs) of the SARS-CoV-2 RNA genome. There are a total of two ORFs in SARS-CoV-2, called ORF1a and ORF1b [16]. A total of 16-NSPs (NSP1-NSP16) are produced from polyproteins, translated from the two ORFs. The viral proteases 3CL and PL pro cleave the polyprotein into NSPs. After cleavage, these proteins assemble and help the virus replicate and transcribe. In addition, the viral genome serves as a template for replication and transcription. The main enzyme involved in SARS-CoV-2 replication and transcription is NSP12, which is an RNA-dependent RNA polymerase (RdRp) [17]. It catalyzes viral RNA genome production and plays a crucial role in the virus’s replication and transcription cycle. This process of replication of the virus by the RdRp enzyme is aided by its co-factors, known as NSP7 and NSP8, working as a complex [18]. NSP12 is thus a primary target for antiviral inhibitors to control its transmission [19]. Beside the RdRp enzyme, other proteins, such as nucleocapsid protein, spike protein, envelope and membrane proteins can also be targeted for drug design [20].

**Figure 1:**
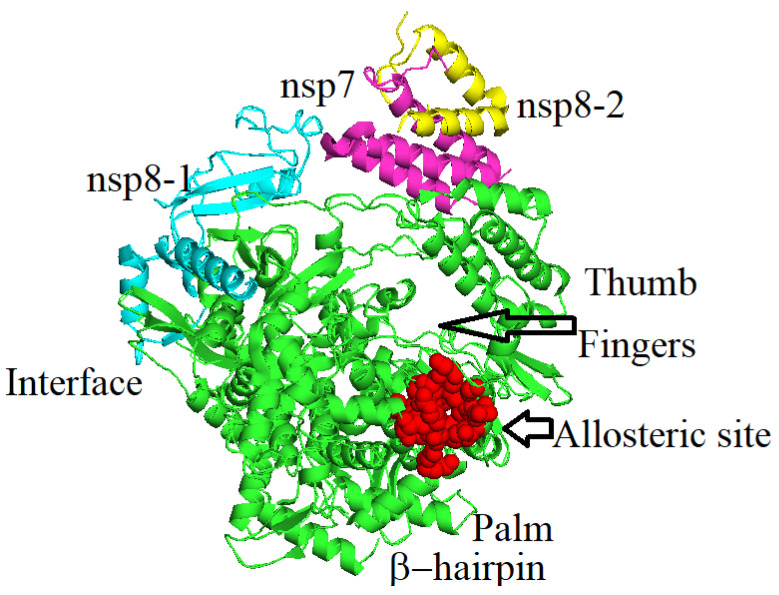
The RNA dependent RNA polymerase of SARS-CoV-2. The red surface shows the identified allosteric site in this enzyme that can be targeted for drug design and inhibition of virus polymerase activity.

The structure of SARS-CoV-2 RdRp has been resolved through cryo-electron microscopy at a resolution of 2.9 Å in complex with its co-factors [21]. The NSP12 enzyme structure contains a “right-handed” domain of RdRp and its residues range from S367 to F920. Its N-terminal extension domain residues range from D60 to R249 and are specific to nidoviruses that assume a nidovirus RdRp-associated nucleotidyl-transferase (Niran)-like organization (Figure 1) [22]. The polymerase domain of SARS-CoV-2 RdRp is made up of three subdomains: a fingers subdomain with residues ranging from L366-A581 and K621-G679, a palm subdomain having residues ranging from T582-P620 and T680-Q815, and a thumb subdomain whose residues are H816-Y920. This follows the viral polymerase family’s conserved architecture (Figure 1) [23]. Catalytic metal ions—found in a variety of viral polymerases that synthesize RNA—are absent in RdRp [24].

Our current approach is based on computational studies of the allosteric site of RdRp of SARS-CoV-2. The binding of molecules to the allosteric site can cause a functional change in the overall structure of the enzyme. The allosteric site can be perturbed by non-covalent molecule binding (e.g., small molecules, ions, or by the binding of an RNA or DNA molecule). Covalent molecule binding can also occur at the allosteric site [25,26]. These modifications in the structure and dynamics of the enzyme may result in catalytic activity being reduced or increased or can change its oligomerization state [27]. SARS-CoV-2 RdRp has a nidovirus RdRp-associated nucleotidyltransferase (NiRAN) domain that helps in the transfer of nucleoside monophosphate to the RNA and catalytic site. It has been observed that the remdesivir triphosphate and other analogues bind not only to the catalytic site but also inside the NiRAN and make allosteric changes in the non-structural protein 12 (NSP12) [26]. It has also been suggested that there are several other pockets inside the RdRp of SARS-CoV-2, where remdesivir triphosphate binds beside the catalytic site and thus inhibits enzyme activity [26,28]. Tian et al. recently reviewed a number of non-nucleoside inhibitors, which have been tested against influenza A and hepatitis C viruses for inhibition of the allosteric site of the RdRp enzymes. In these two viruses, the allosteric sites are mostly present in various thumb and palm domains of the RdRp. Several of these non-nucleoside inhibitors have reached phase III clinical trials [29]. The non-nucleoside inhibitors are less toxic and have lower side effects as compared to the nucleoside inhibitors. It is therefore important to investigate non-nucleoside inhibitors that target the allosteric site of the RdRp of SARS-CoV-2. In a recent in silico study on the RdRp of SARS-CoV-2, it was observed that different ligands bind very well within various pockets of the thumb, palm I and palm II domains [30].

The current research investigates the inhibitory role of flavonoids against RdRp of SARS-CoV-2 [31]. They are non-nucleoside inhibitors of numerous viruses and have a wide range of antiviral activities [32,33,34,35]. Our approach is to find inhibitors from this family of phytochemicals, which can specifically act on the allosteric pocket of RdRp enzyme of SARS-CoV-2 by utilizing various computational techniques, such as molecular docking and simulation. These findings may aid in the development and advancement of new SARS-CoV-2 viral drugs and kick-start the drug discovery process.

## 2. Results and Discussion

### 2.1. Molecular Docking, Binding Energy and Interaction Analysis of Docked Flavonoids against SARS-CoV-2 NSP-12 RdRp-Enzyme’s Allosteric Site

The flavonoids with the lowest binding affinity energy (S-Score), lowest RMSD refine score and best interactions with RdRp (NSP12) of SARS-CoV-2 in complex with other co-factors will be discussed here (Table 1 and Table 2 and Figure S1 and Table S1). The top three lead candidates with the lowest S-Score, lowest RMSD and most interactions with the allosteric site residues were further selected for MD-simulations studies.

#### 2.1.1. Interaction Analysis of Naringoside

Naringoside flavonoid showed a total of nine interactions with the allosteric site residues of the target protein. There were eight hydrogen bond interactions and one interaction of the arene-cation type between the flavonoid nucleus ring-A and Arg583 of the allosteric site. The –OH group present at the ring-A made a hydrogen bond interaction with allosteric site residue Ser592. Furthermore, the two-glycoside moieties attached with the ring-A of the naringoside flavonoid formed the remaining seven H-bonds with the allosteric site amino acid residues. The –OH hydroxyl groups of the glycosides interacted with Thr604, Gly584, Thr582, Arg583, Met601, Thr582 and Ala585 of allosteric site residues. The binding affinity energy (S-Score) of naringoside with the allosteric site of the target enzyme was −19.0 kcal/mol (Table 1 and Table 2).

#### 2.1.2. Interaction Analysis of Aureusidin 4,6-Diglucoside

Aureusidin 4,6-diglucoside flavonoid belongs to the aurone class of flavonoids. It showed six interactions with the allosteric site residues of the protein. The six interactions of this flavonoid were all hydrogen bond interactions. The ketone group on ring-C of this aurone flavonoid engaged with Thr604 of the allosteric site via H-bonding. Thr604 also formed a H-bond with the ring-B of the aurone flavonoid nucleus. Arg750 and Thr586 also made two H-bonds with –OH groups of the ring-B of this flavonoid. The two-glycoside moieties attached to the ring-A of this flavonoid resulted in two interactions with allosteric site residues, Phe594 and Asn600. The individual glycosides each make single H-bonds via –OH groups. The binding affinity energy (S-Score) of this flavonoid was −19.21 kcal/mol (Table 1 and Table 2).

#### 2.1.3. Interaction of Myricetin

This flavonoid exhibited good binding energy (S-Score) of −18.17 kcal/mol. It also exhibited good interactions with the allosteric site residues; five interactions were seen and one of the interactions was an arene-cation interaction of the ring-A of the main nucleus of the flavonoid with Arg583. The ring-A also made a H-bond via its attached –OH group with Ser592. The ring-B hydroxyl group also formed a H-bond with the Thr604 allosteric amino acid, and the ring-C of this flavonoid engaged in two H-bonding interactions with Met755 and Gly584 via its two –OH groups at 4′ and 5′ positions (Table 1 and Table 2). Myricetin can also bind tightly within the active site of the RdRp of SARS-CoV-2, which was also confirmed from MD simulation [36].

In similar fashion, plant-based polyphenols and flavonoids were used against the active site of SARS-CoV-2 RdRp. These flavonoids showed lower free energy of binding as compared to our results. This difference in free energy may be due to the utilization of different software having different force field and mathematical equations for the calculation of binding free energy [36]. Mostly, these docked flavonoids are also docked in this study but the target here is the allosteric site instead of the active site of RdRp [36]. Experimental results also showed that flavonoids, such as baicalin and baicalein, inhibit the SARS-CoV-2 3CLpro and PLpro protease enzymes in micromolar concentration [37]. The myricetin blocks the SARS-CoV-2 3CL protease through covalent inhibition and baicalein blocks through non-covalent inhibition [38,39]. The crystal structure of myricetin and baicalin in complex with SARS-CoV-2 protease has also been resolved at high resolution. These observations showed the important inhibitory role of flavonoids against viruses.

### 2.2. MD Simulation Analyses

MD simulations were used to evaluate the target protein and ligand-binding stability, and their dynamic properties during the virtual simulation time. The RMSD fluctuation was computed separately for both the protein and ligand complexes in the MD simulations, and it was observed to be within 3 Å. The existence of intermolecular interactions among ligand and RdRp was monitored during the study using the simulation interaction diagram tool implemented in the Desmond MD package [40]. The simulation was performed for all three top hit flavonoids and the RMSD plot of the complexes produced during the MD-simulation revealed stable binding of the ligands with SARS-CoV-2 RdRp.

#### 2.2.1. MD Simulations Stability Analysis of RdRp-Apo-Enzyme

**Figure 2:**
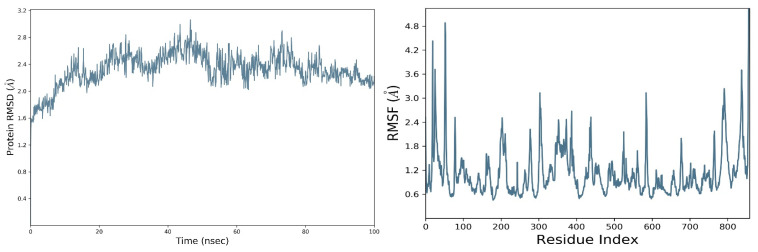
The root mean square deviation (**Left panel**), and root means square fluctuation (**Right panel**) of the amino acid residues of RdRp apo-enzyme of SARS-CoV-2.

RdRp in apo form was simulated for 100 ns of virtual time and RMSD calculated. RMSD designates how much the protein deviates from its initial resolved structure. At the start, it remained very low, between 1.6 and 2.0 Å, but at about 15 ns it fluctuated to 2.5 Å, then stabilized a bit and increased again to 3 Å at 50 ns time. After about 60 ns of the simulation, it stabilized and remained at about 2.4 Å. This fluctuation further stabilized and was found to be 2.1 Å at the end of the simulation, indicating that the protein stabilized and attained equilibrium and remained uniform in this form along the trajectory. An RMSD of 2.1 Å seems very stable for this protein (Figure 2).

Moreover, the root mean square fluctuations (RMSF) stability studies showed that at the start and the end of the protein chain it fluctuated up to 4.8 Å, suggesting that the terminal regions of the protein are quite flexible. These sites in the protein chains have relatively high flexibility compared to the internal regions of the protein, so these kinds of fluctuations are normal in proteins. It can also be noted that the allosteric site of the protein, which lies between 500–800th residues, was stable and its RMSF values remained between 0.6 and 1.2 Å (Figure 2 right panel). The complexed ligated form of the protein in the simulations was further stabilized by the attachment of flavonoid ligands, which are discussed in the upcoming protein–ligand complex simulations.

Furthermore, the secondary structure element studies of RdRp demonstrated that our protein had a lot of helix regions. These helices are very important as they contribute to the stability of secondary structures of the protein. These regions are H-bond rich regions, which means that the H-bonds between the amino acids of the protein can provide more stability to the protein structure. The linear strand regions were low in the protein, which means that it is more compact and not linear due to the presence of these helices. In Figure 1 of SSE, brown spikes represent helices and the blue spikes are linear strand regions while the white color regions are loops in the RdRp apo-enzyme. Figure S2 shows the SSE total content elements of each amino acid residue, while the protein helices, loops and strands are shown as a function of the protein residue index in Figure S3.

#### 2.2.2. Simulation of Aureusidin 4,6-Diglucoside and RdRp Complex

**Figure 3:**
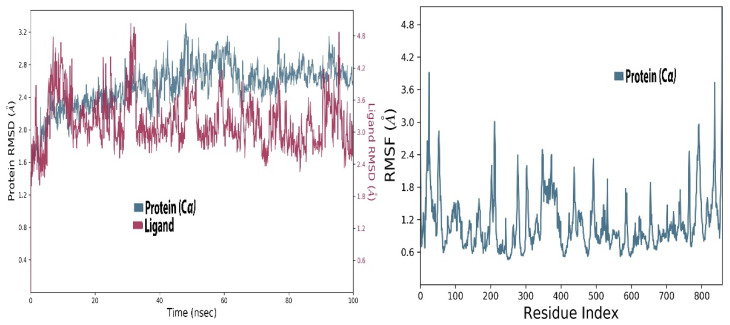
Root mean square deviation (**Left panel**) and root mean square fluctuation (**Right panel**) of RdR in complex with aureusidin 4,6-diglucoside.

In a 100 ns simulation of aureusidin 4,6-diglucoside and RdRp complex, the RMSD was low to begin with but later increased slowly. The RMSD of this complex is stable between 60 to 65 ns, indicating that at the attainment of equilibrium, it remained constant up to the end of 100 ns of simulation, and its RMSD was close to 2.4 Å. The ligand in complex started at a lower RMSD’s apo-protein, but at 15 ns and 30 ns it fluctuated and then again stabilized. The RMSD is lower at the end of the 100 ns time and a further decrease indicates the stabilization of the ligand. The RMSD of the aureusidin 4,6-diglucoside and RdRp-enzyme complex during the simulation was on average about 2.8 Å, which is lower than the 3 Å of the apo-protein. This shows stable association of ligand–protein during the 100 ns (Figure 3). As the current simulation was carried out for only 100 ns, it is unclear whether the complexes are stable for micro- and milli-seconds. The present results suggest that the drug molecules tested are stably bound to RdRp for at least 100 ns, raising the possibility that these molecules could be candidates as potential drugs targeting RdRp.

It can also be observed in Figure 3 that the RMSD of the ligand remained in the range of 2.4–3.6 Å, which is stable. Its radius of gyration (rGyr), which is equal to its principal moment of inertia, measures the ‘extendedness’ of a ligand, and was high at the start but stabilized in the end. Furthermore, the solvent accessible surface area (SASA), polar surface area (PSA) and the van der Waals surface area (MolSA) was high (Figure S4). In the RMSF plot, one can notice that amino acids 200–215 fluctuated up to 3 Å. The fluctuation of the residues at the end of the protein chain was higher; these higher RMSF values suggest that the loop regions present in the enzyme have more conformational adaptability and are instigating these fluctuations. While the RMSF values of the whole protein did not fluctuate much and had an average RMSF of 1 Å at the allosteric residues, this site of the protein persisted to be stable during the 100 ns simulation, suggesting that the ligand and protein was in stable form. The RMSF of the protein remained between 0.6 to 1.4 Å (Figure 3).

The simulation data were post-processed by interaction analysis to investigate the crucial protein–ligand-binding details. Aureusidin 4,6-diglucoside was in constant contact with the allosteric site residues (i.e., Asn600, Arg583, Ser592, Phe594, Thr604, Met755) and interacted throughout the 100 ns simulation, validating the docking results, and demonstrating that these allosteric site amino acids exhibited strong association with this ligand. As observed in the docking studies, Arg583, Ser592 and Asn600 also showed strong binding association for the duration of the 100 ns simulations studies (Figure S5). It can also be observed in the interaction fraction diagram (Figure S6) that the allosteric site residues in the MD-simulation had various types of interactions, such as those also observed in the docking studies. They are strong interactions and are present throughout the 100 ns simulation (Figures S6 and S9). The most active allosteric residues, Arg583, Ser592, Phe594, Asn600 and Asp608, exhibited H-bonds and water bridges interactions throughout the simulations, suggesting that the ligand was in strong and stable association with the RdRp-enzyme. The interaction of the ligand can be observed with the allosteric residues during MD- Simulations in Figures S5 and S6. The rotational flexibility of the aureusidin 4,6-diglucoside and the rotations of various bonds in the molecule, which are presented in the torsional angle diagram, where full rotations of certain bonds were noticed in one of the glucose subunit, while the flavonoid and the second sugar molecule was rigid (Figures S7 and S8). These fluctuations are because of the flexibility and can be considered normal; it can be observed that the RMSD of the ligand was low, which is a sign of stability. The allosteric site in the palm region of RdRp of SARS-CoV-2 was also investigated by others; various nucleoside and non-nucleoside inhibitors bind in this region [30]. From Figure 2 and Figure 3, the RMSF of free protein and protein in the complex with ligand, are compared, then the fluctuation in several residues from 500–800 region is decreased. This lower dynamic movement in several domains and in the catalytic site is due to the allosteric effect, which resulted from the binding of ligand to the allosteric site. The catalytic site of the RdRp of SARS-CoV-2 is present in the palm subdomain part and made of motif A-G [41]. This active site comprises D618, D760 and D761, and other residues from various motifs that include T680, T710, F753, N767, L775, E796, H810, V820, K912, E921, K500 and S518 [41].

#### 2.2.3. MD Simulations of Myricetin and RdRp Complex

**Figure 4:**
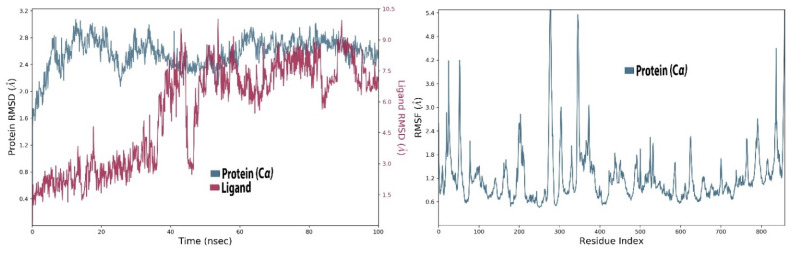
The left panel shows the RMSD of C-alpha of amino acid residues of RdRp (blue) or myricetin (maroon) during 100 ns of simulation time. The right panel shows fluctuation in the amino acid residues of RdRp in complex with myricetin.

Stability studies of this complex demonstrated that during simulations, the RMSD of the protein at the starting time fluctuated and reached about 2.8 Å from 0–30 ns time of simulation. After the 30 ns mark, the protein stabilized, indicating that it reached equilibrium and had an average RMSD of about 2.4 Å at the end of the 100 ns (Figure 4). The ligand fluctuated too much, but at 40 ns it converged and stabilized a bit and then again fluctuated but at about 50 ns it stabilized again; however, compared to the protein, its RMSD was higher.

It can be observed in the RMSF diagram that, similarly to the first complex, the protein RMSF fluctuated at the start and end of protein chain residues. A maximum fluctuation of about 5 Å was noted at about 300 residues, which is possibly caused by loop regions, which are comparatively more flexible than the other structures present in the protein. The ligand-binding allosteric residues did not fluctuate and had stable average RMSF of 1.3 Å, suggesting that the ligand stabilized the protein in complex bound form (Figure S10). It can be observed in Figure 4 that the ligand RMSD was about 0.4 Å in the 15–38 ns time range, fluctuated to 1.3 Å after 40 ns, stabilized to 0.3 Å and at the end it again increased to about 1.2 Å. These fluctuations in RMSD are fairly stable, as it did not cross the 3 Å threshold. The radius of gyration of myricetin remained at 3.75 Å and after 100 ns it further contracted and came down below 3 Å. Furthermore, the SASA values were on average 80–90 Å^2^; Polar Surface Area (PSA) and the van der Waals surface area (MolSA) were relatively low compared to the first complex, indicating that myricetin was buried in the allosteric site pocket during the simulation (Figure S11).

It was observed that certain allosteric residues exhibited stronger interactions with myricetin at 40 ns of virtual time but later did not interact with the ligand. However, as observed in the docking studies, the main interacting amino acids, i.e., Arg583, Gly584, Ser592 and Asn600, exhibited stable and strong interactions along the 100 ns simulation trajectory up to the end (Figure S12). These interactions also influence the catalytic site of RdRp, as observed from the fluctuation of various residues in the RMSF graph. Binding in the allosteric site will modify the active site of the enzyme and its affinity for its own catalyzing reactants will be lowered. In the interaction fraction histogram (Figures S11 and S12), it can be observed that the allosteric site residues exhibited contacts of various types with the ligand in the complex. The most active interacting amino acid observed in the docking process—Arg583 (discussed earlier)—was the most active during the 100 ns simulation and exhibited hydrogen bond and hydrophobic interactions with the ligand. The other most active amino acids were Thr591, Ser592, Asp608 and Met755. These amino acids were observed to have exhibited strong bonding along the 100 ns trajectory with the myricetin in complex with the RdRp-enzyme. Myricetin was flexible in the allosteric site and torsional rotations of the –OH side groups of the ligand were also noted along the simulation trajectory (Figures S13 and S14). The myricetin also inhibited the activity of the SARS-CoV-2 active site. Thus, these flavonoids can interact with both the allosteric and active site, such as the remdesivir triphosphate and other related nucleoside inhibitors [26,36]. Overall, these computational studies offer information that not only the catalytic site in the RdRp should be targeted for drug design but allosteric sites are equally important for inhibition through various potential ligands [28].

#### 2.2.4. MD Simulations of Naringoside in Complex with RdRp-Enzyme

**Figure 5:**
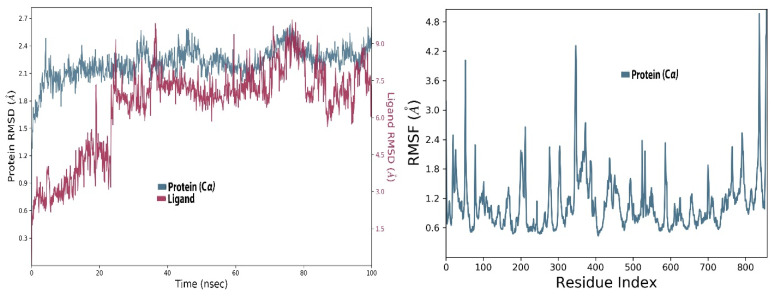
Left panel demonstrates the RMSD of C-alpha of amino acid residues of RdRp (blue), while the maroon color is that of the naringoside flavonoid during the 100 ns simulation time. The right panel showed the fluctuation of C-α carbon of the amino acid residues of the RdRp in complex with naringoside.

The RMSD of the protein rise a little during simulations and reached about 2.5 Å at 0–30 ns time of simulation, but later the protein stabilized and had an average RMSD value of about 2.3 Å, which was below the limit of 3 Å, suggesting that it attained equilibrium (Figure 5). At 25 ns, the ligand’s RMSD converged and stabilized with the protein. The ligand’s RMSD was a bit higher than that of the protein in the complex.

The RMSF diagram shows that similarly to the first complex, the protein RMSF fluctuated at the start and ends of the protein chain residues, which was a maximum fluctuation of about 4 Å observed by the amino acid residue number 350 in the protein chain. This was likely caused by the loop regions, which are comparatively more elastic and flexible than the other secondary structures present in the protein, resulting in higher RMSF at these sites. However, the ligand-binding allosteric site’s RMSF values did not fluctuate and had a constant average RMSF value of about 0.3 Å, indicating that the ligand stabilized the protein in complex bound form (Figure 5). Moreover, the RMSD of the naringoside can be seen at the start to have a value of 2.5 Å in (Figure 5) and rises continuously till 25 ns and have RMSD of 7 Å. The radius of gyration (rGyr) of RdRp in complex with nariginoside was about 4.9 Å average along the 100 ns simulation time. Furthermore, the SASA values were on average 190 Å^2^, and the polar surface area (PSA) and the van der Waals surface area (MolSA) were high (Figure S15).

In the protein–ligand interaction studies during the simulations, they were observed to be the same, as were the case with the other two complexes. The interactions observed during the docking studies of narigoside were observed here also confirming that the allosteric site residues Arg583, Ser592, Asn600 and Thr604 were in constant contact with the ligand along the 100 ns trajectory (Figure S16). These interactions also affect other domains, especially the active site inside the RdRp enzyme, through allosteric modifications. The histogram interaction fraction showed that the allosteric site residues made different types of contacts with the ligand in the complex form. The most active interactive amino acid observed in the docking process was Arg583. It was also the most active during the 100 ns simulation and displayed hydrogen bond and hydrophobic interactions with the ligand. The other most active amino acids were Thr591 and Asn600. These results confirmed that the ligand was in contact with the protein throughout the simulation time and were stable in the target protein allosteric site (Figure S17). The Figures S18 and S19 demonstrates the torsional rotational angles of the naringoside that help in various interactions between the ligand and the protein.

### 2.3. In Silico Prediction of Absorption, Distribution, Metabolism and Excretion (ADME), and Toxicity Studies

In silico ADME and toxicity properties of the top three hits were evaluated with the admetSAR online server [42]. Of the top three flavonoids, myricetin had the highest enhanced human intestinal absorption (HIA) score followed by the naringoside and aureusidin 4,6-diglucoside flavonoid. When administered orally, a compound with a higher HIA is more likely to be absorbed from the gastro-intestinal tract. Aureusidin 4,6-diglucoside flavonoid had the highest blood–brain barrier (BBB) absorption, whereas the other two flavonoids were negative for BBB. When it comes to estimating P-glycoprotein (P-gp) discharge of these three flavonoids, all of them were substrates of this protein and did not inhibit the P-gp.

None of the flavonoids inhibited the secretion of drugs into urine by renal-organic cation-transporter proteins. In metabolism studies, it was discovered that myricetin was a non-substrate and non-inhibitor of all other CYP450 microsomal enzymes, but that it only repressed CYP450-1A2 and 3A4, while the two other flavonoid molecules were neither substrates nor inhibitors of CYP450. The term “noninhibitor of cytochrome p450 enzymes” refers to a compound that does not interfere with the biotransformation of substances metabolized by the cytochrome p450 enzyme. The AMES toxicity test is often used to see if a substance is mutagenic, or if it induces congenital fetal defects during pregnancy. Myricetin was found to be non-AMES toxic while the aureusidin 4,6-diglucoside and naringoside were AMES + toxic. None of the three flavonoids were shown to be carcinogenic. In the oral acute toxicity in silico evaluations, it was found that myricetin has LD_50_ values greater than 50 mg/kg but less than 500 mg/kg and is listed in category-II by the US-EPA. Whereas the other two flavonoids are listed as category-III, which includes compounds and substances with LD_50_ concentrations larger than 500 mg/kg but less than 5000 mg/kg, while category-I substances are the most toxic substances.

Lethal dosage or LD_50_ in silico toxicity (acute toxicity) in the rat model revealed that myricetin had LD_50_ = 3.02 mol/kg, while aureusidin 4,6-diglucoside and naringoside had LD_50_ of 2.65 and 2.26 mol/kg values, respectively. These have higher LD_50_ values, which means that these flavonoids can be acceptable and well-tolerated when consumed (Table 3). Based on several of these computational observations, aureusidin 4,6-diglucoside is the most promising candidate for future experimental studies.

In the last two decades, we have seen the emergence of several viral pandemics across the globe like Ebola, SARs, MERs, ZIKV and SARS-CoV-2 [4,43,44]. To control the spread of SARS-CoV-2 and other zoonotic viruses, it is important to use a multi-pronged strategy. Besides vaccine development, different drug inhibitors for viral enzymes need to be developed using modern medicinal chemistry techniques. Antiviral proteins, such as interferons and zinc finger antiviral proteins, need to be further studied to better understand the human immune response [45]. In this way, the scientific community will be able to control the deadliest strains of continuously emerging viruses.

## 3. Methodology

### 3.1. Selection of Target Protein and Prediction of Its Allosteric Site

The 2.9 Å resolution structure of the RdRp with PDB identification number 6m71 was obtained from the RSCB protein data bank [21]. This PDB file was then checked for allosteric sites through an online server, Allosite Pro 2016 (http://mdl.shsmu.edu.cn/AST/module) (accessed on 3 December 2020) [46]. After the prediction of the allosteric site by Allosite Pro (2016), the protein PDB file with the predicted site was downloaded and its allosteric site viewed via Pymol Molecular Graphic Visualizer software 2.4 [47]. The allosteric site residues highlighted via Pymol were noted for further computational studies.

The allosteric site residues identified by Allosite Pro server were all present in the Chain-A of the RdRp and the predicted allosteric site residues identified had the following amino acid residues in the pocket. The sequence and types of the amino acid residues identified are: Thr582, Arg583, Glt584, Ala585, Thr586, Val587, Val588, Thr591, Ser592, Gly597, Asn600, Met601, Thr604, Val605, Arg750, Ser754, Met755 and Met756. This allosteric site is not yet validated through experiments.

### 3.2. Ligand Preparation

Prior to docking studies, a literature study was performed for the selection of ligands (flavonoids). We have selected most of the flavonoids based on their previous use as antiviral agents in both experimental and computational studies. This is the main reason we have tried these inhibitors in our docking and simulation study of RdRp of SARS-CoV-2. A total of one hundred flavonoids were selected for docking studies, and the structures of these selected flavonoids were prepared using ChemDraw software 10.0 [48] for the molecular docking.

### 3.3. Molecular Docking

Docking of the selected flavonoids with SARS-CoV-2 RdRp was performed through Molecular Operating Environment (MOE; Chemical-Computing-Group) using the established protocols [49,50,51,52]. The missing atoms and residues in the SARS-CoV-2 RdRp enzyme were added through MOE structure modeling. SARS-CoV-2 RdRp protein was prepared for docking by the addition of hydrogen atoms using the 3D-protonation module of MOE software; the partial charges were also corrected for the protein. RdRp was energy minimized via the Amber-99 force field present in MOE. After these necessary preparations, we then utilized the in-built site finder MOE-Module for locating the allosteric site, which was earlier predicted by Allosite-Pro online server [46]. The allosteric site residues earlier noted by using Pymol software were used to identify and locate our protein allosteric pocket via the site finder function of MOE. In MOE, the site finder module identifies a number of binding pockets and presents them in a sequence with their amino acid residues. As we have identified the allosteric site before through Allosite-Pro, in the MOE results the binding pocket that has similar residue, such as Allosite, was selected as a binding pocket [46]. For docking of the ligands with our protein, London-dG scoring functions were used for binding energy calculations, as performed previously [50,51,52,53]. Prior to molecular docking, the structure of ligands were also energy minimized in MOE [49]. For each tested flavonoid molecule, the MOE software produced around 20 different placements, called poses. Among these poses, the pose with the highest number of interactions between RdRp enzyme and the flavonoid ligand was recorded. MOE provides a numerical value for the interaction of ligand to protein in the form of S-score. MOE shows all the possible interactions made during the docking; these include salt bridges, hydrogen bonding, cation-π and hydrophobic interactions, etc.

### 3.4. MD Simulation

The top three lead flavonoids with the best interactions, lowest docking score (S-score) and lowest root-mean-square-deviations (RMSD) calculated via MOE and the apo-enzyme were then used for further computational studies via Schrodinger’s Desmond module MD-Simulation Software. For MD-Simulations, the best docked top three flavonoids in complex with the SARS-CoV-2 RdRp were saved in PDB format in MOE and then used for further stability studies in Schrodinger’s Desmond module [54]. The Desmond in-built system Builder tool was used to create the solvated water-soaked MD-Simulation system. The TIP3P model were the solvating model in the experiment. An orthorhombic box created at least 10 angstrom from the protein’s outer surface. The simulation systems were neutralized by the addition of a sufficient amount of counter ions. The isosmotic state was preserved by adding 0.10 mol/L sodium and chloride ions to the simulation panel. Before the simulation, a pre-defined equilibration procedure was carried out. The system was equilibrated using standard Desmond protocol at constant pressure and temperature (NPT ensemble, 300 K, 1 bar) and the Berendsen coupling protocol with one temperature group. Hydrogen atom bond length was constrained using the SHAKE algorithm. Particle Mesh Ewald (PME) summation method was used to model long-range electrostatic interactions. A cut off of 10 Å was assigned for van der Waals and short-range electrostatic interactions. The MD simulation was run at ambient pressure conditions of about 1.013 bar while the temperature was set to 300 K for 100 nano-second MD-Simulation, and 1000 frames were stored into the simulation trajectory file. The simulation run time for each complex system and Apo system was 100 ns in total. After simulations, stability studies of the complex and Apo system were performed (i.e., RMSD, RMSF).

### 3.5. ADME and Toxicity Prediction

Computational studies involving the metabolism, absorption, toxicity and distribution of the top three selected flavonoids in a living system were performed through the admet-SAR online server (http://lmmd.ecust.edu.cn/admetsar2) (accessed on 10 March 2021) [42].

## 4. Conclusions

We screened flavonoid phytochemicals against RdRp and utilized a novel approach of targeting the allosteric site of this enzyme. Various in silico computational tools were employed to perform these computational studies, including molecular docking, simulation and ADMET studies. Several studies have reported antiviral activities of these phytochemicals. In this computational work, it was found that they bind to the allosteric site with strong affinities, and the binding was also confirmed by molecular dynamics simulations. They make stable to semi-stable associations and their interactions were the same as seen in the molecular docking studies. The three flavonoids naringoside, myricetin and aureusidin 4,6-diglucoside showed maximum interactions with the lowest binding energies. Arg583, Thr604, Asn600 and Ser592 of the allosteric site of RdRp were confirmed via MD-simulations to interact strongly with the top three selected flavonoids through both H-bonding and hydrophobic interactions during 100 ns virtual simulation time. Moreover, ADMET prediction studies also confirmed that these flavonoids have ample bioavailability because of good HIA values; they were all also non-carcinogenic and non-toxic. The SARS-CoV-2 enzyme active site and surface proteins are mutating at high rates; therefore, exploring and targeting the allosteric sites, as performed in this research, can result in more potent novel drugs that can effectively bind and repress viral activity. In this computational research of allosteric site inhibition, the top hits identified exhibited strong binding and interactions with the allosteric site of RdRp polymerase. From this, one may assume that these identified phytochemical flavonoids can be potential drug candidates against the coronavirus.

## Figures and Tables

**Table 1:** Chemical structures, 2D and 3D images of the top three flavonoids interacting with the allosteric site residues.

S/N	Flavonoid Chemical Structure	2D Image of the Flavonoid Interacting with the Allosteric Site	3D Image of the Flavonoid Interacting with the Allosteric Site
1	* 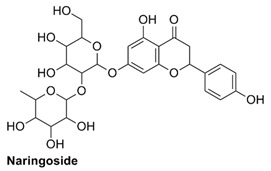 *	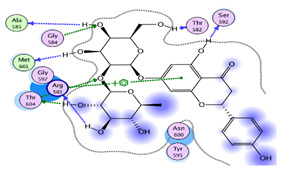	* 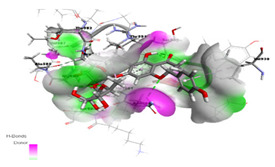 *
2	* 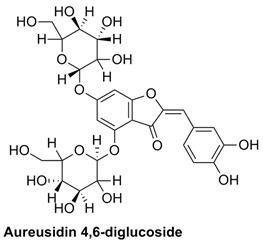 *	* 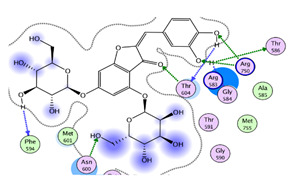 *	* 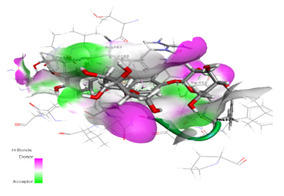 *
3	* 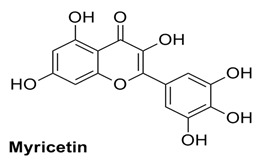 *	* 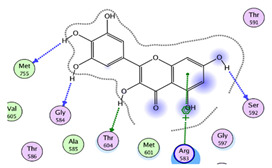 *	* 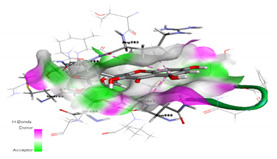 *

**Table 2:** Flavonoids, which we screened against the RdRp enzyme allosteric site. The columns show the binding affinity energy (docking S-Score), and RMSD refine values calculated during molecular docking. The number and types of interaction they formed with the allosteric site amino-acid residues are also presented.

S/N	Flavonoid	Docking S-Score	Interactions Formed	Residues Involved in Interactions
1	Naringoside	−19.00 kcal/mol	1 Arene-Cation 8 H-Bonds	2 + Arg583, 2 + Thr604, Met601, Ala585, Ser592, Thr582.
2	Aureusidin 4,6-diglucoside	−19.21 kcal/mol	5 H-bonds	2 + Asn600, Met755, Gly584, Ser592
3	Myricetin	−18.17 kcal/mol	1 Arene-Cation 4 H-Bonds	Met755, Gly584, Thr604, Arg583, Ser592

**Table 3:** Various ADMET profile in silico predicted values for the selected top three flavonoids.

S/N	Flavonoid	LD_50_ Rat Model	BBB	Carcinogens	HIA	Cytochrome p450 Inhibition/Substrate	Oral Acute Toxicity	AMES Toxicity
1	Myricetin	3.02 mol/kg	0.5711	No	0.9650	Non-substrate and Only Inhibited CYP450-1A2 and 3A4.	Category-II	No
2	Naringoside	2.26 mol/kg	0.8414	No	0.8645	Non-substrate/Non-inhibitor	Category-III	Yes
3	Aureusidin 4,6-diglucoside	2.65 mol/kg	0.5417	No	0.7082	Non-substrate/Non-inhibitor	Category-III	Yes

## Data Availability

Data will be provided upon request.

## Supplementary Materials

The following supporting information can be downloaded online, Table S1. The docked flavonoid molecule with binding free energy, types of interaction with RdRp enzyme, and residues involved in interaction with the ligand. Figure S1. The docked flavonoid molecules in 2D and 3D structure inside the allosteric binding pocket. Figure S2. Percentage of the secondary structure elements of the residue of the apo-enzyme. Figure S3. Secondary structure elements residue index of apo-protein as a function of simulation time. Figure S4. The polar surface area (PSA); solvent accessible surface area (SASA), and the van der Waals surface area (MolSA) of the RdRp in complex with aureusidin 4,6-diglucoside. Figure S5. Contacts of the different residue and the ligand during the 100 ns simulation time. Figure S6. Various types of interaction made by the amino acid residues of the protein during the simulation.; Figure S7. Torsion and flexibility in the aureusidin 4,6-diglucoside. Figure S8. Torsional angles of the aureusidin 4,6-diglucoside flavonoid. Figure S9. Various types of interaction made by the aureusidin 4,6-diglucoside with the amino acid residues of the protein during the simulation. Figure S10. Polar Surface Area (PSA), solvent accessible surface area, the van der Waals surface area, (MolSA), radius of gyration and RMSD of the RdRp in complex with the myricetin during 100 ns simulation. Figure S11. Allosteric residues contacts of the RdRp with the myricetin flavonoid ligand. Figure S12. Various types of interactions made by specific amino acids with the myricetin flavonoid. Figure S13. Myricetin torsions that are involved in its fluctuation inside the protein. Figure S14. Torsional angles of the myricetin. Figure S15. Polar surface area, solvent accessible surface area, the van der Waals surface area, radius of gyration and RMSD of the RdRp in complex with the naringoside during 100 ns simulation. Figure S16. Number of contacts made by the allosteric and other residues of RdRp. Figure S17. Various types of interactions and its total fraction made by the allosteric residues with ligand. Figure S18. Torsion angles of the naringoside ligand. Figure S19. Torsional angles of the narigoside.

## Sample Availability

Samples of the compounds are not available from the authors.
