# Supplementary material for: Computational Study of SARS-CoV-2 RNA Dependent RNA Polymerase Allosteric Site Inhibition
Source: Molecules. 2021 Dec 30;27(1):223. doi: 10.3390/molecules27010223 (PMC8746673; doi:10.3390/molecules27010223)
Supplement: Supplementary file 1 [file molecules-27-00223-s001.zip › molecules-1435865-supplementary.pdf]

Supplementary Material

Computational Study of SARS-CoV-2 RNA Dependent RNA Polymerase Allosteric Site Inhibition

Shah Faisal [1], Syed Lal Badshah [1,*], Bibi Kubra [1], Mohamed Sharaf [2,3], Abdul-Hamid Emwas [4], Mariusz Jaremko [5,*] and Mohnad Abdalla [6,*]

[1] Department of Chemistry, Islamia College University Peshawar, Peshawar 25120, Pakistan; faisalvbs@gmail.com (S.F.); kubrakha621@gmail.com (B.K.)
[2] Department of Biochemistry and Molecular Biology, College of Marine Life Sciences, Ocean University of China, Qingdao 266003, China; mohamedkamel@azhar.edu.eg
[3] Department of Biochemistry, Faculty of Agriculture, AL-Azhar University, Nasr City, Cairo 11751, Egypt
[4] Core Labs., King Abdullah University of Science and Technology (KAUST), Thuwal 23955-6900, Saudi Arabia; abdelhamid.emwas@kaust.edu.sa
[5] Smart-Health Initiative (SHI) and Red Sea Research Center (RSRC), Division of Biological and Environmental Sciences and Engineering (BESE), King Abdullah University of Science and Technology (KAUST), Thuwal 23955-6900, Saudi Arabia.
[6] Key Laboratory of Chemical Biology (Ministry of Education), Department of Pharmaceutics, School of Pharmaceutical Sciences, Cheeloo College of Medicine, Shandong University, 44 Cultural West Road, Jinan 250012, China
* Correspondence: sbadshah@icp.edu.pk (S.L.B.); mariusz.jaremko@kaust.edu.sa (M.J.); mohnadabdalla200@gmail.com (M.A.)

Table S1. The docked flavonoid molecule with binding free energy, types of interaction with RdRp enzyme, and residues involved in interaction with the ligand.

S/N	Flavonoid	Docking S-Score	Interactions Formed	Residues Involved in Interactions
1	(*Z*)-2-((1-butyl-1H-indol-3-yl)methylene)-4,6-dihydroxybenzofuran-3(2H)-one	−13.12 kcal/mol	1 Arene-Cation interaction	Arg583
2	4',5,6,7-Tetramethylflavone	−11.66 kcal/mol	None	-
3	5,7,30 ,40 -tetra-*O*-methylquercetin	−13.32 kcal/mol	1 Arene-Cation<br>1 H-bond	Arg583, Asn600
4	5-Hydroxy-3,3',4',6,7,8-hexamethylflavone	−12.96 kcal/mol	1 H-Bond	Asn600
5	6,7-Dihydroxyflavone	−11.02 kcal/mol	2 H-Bonds	Asn600, Thr604
6	L-Epicatechin	−15.01 kcal/mol	1 H-Bond	Asn600
7	6-Chloro-3-methoxy-4'-oxazolinylflavone	−11.26 kcal/mol	None	-
8	6-Chloro-4'-oxazolinylflavanone	−10.66 kcal/mol	1 H-Bond	Asn600
9	6-Chloro-4'-oxazolinylflavone	−11.58 kcal/mol	None	-
10	3,5,7,2'-Tetrahydroxyflavone	−11.26 kcal/mol	1 H-Bond	Asn600
11	PubChem-CID: 6481172	−10.39 kcal/mol	None	-
12	7''-methylamentoflavone	−13.39 kcal/mol	2 H-Bonds	With Thr604
13	Amentoflavone	−15.83 kcal/mol	None	-
14	Apigenin	−10.97 kcal/mol	1 H-Bond	Asn600

15	Astilbin	−13.76 kcal/mol	1 H-Bond<br>1-Arene-Cation	Arg583, Asn600
16	Baicalein	−12.70 kcal/mol	1 H-Bond	Asn600
17	3,7,4'-Trihydroxyflavone	−10.56 kcal/mol	1 Arene-Cation<br>3 H-bonds	Met755, Ser592, Arg583, Thr604
18	Biochanin A	−12.00 kcal/mol	1 Arene-Cation	Arg583
19	Cianidanol	−12.76 kcal/mol	None	-
20	DihydroQuercetin	−12.75 kcal/mol	None	-
21	Diosmin	−14.35 kcal/mol	4 H-Bonds	Tyr595, Arg583, Ser592, Thr604
22	PubChem-CID 3086603	−17.29 kcal/mol	4 H-Bonds	2 + Asn600, Thr582, Val587, Ala585
23	Epicatechin Gallate	−14.57 kcal/mol	1 Arene-Cation	Arg583
24	Eriocitrin	−14.14 kcal/mol	2 H-Bonds	Ala585, Arg583
25	Eriodictyol 7-glucuronide	−18.09 kcal/mol	4 H-Bonds	Met755, Tyr595, Asn600, Gly584
26	Tetrahydroxyflavanone	−12.63 kcal/mol	1 H-Bond	Ser592
27	Fustin	−12.69 kcal/mol	1 Arene-Cation<br>4 H-Bonds	Arg583, Met601, Thr604, Ala585
28	Gabranine	−11.29 kcal/mol	None	-
29	Galangin	−13.69 kcal/mol	1 Arene-Cation<br>2 H-Bonds	Arg583, Thr604, Ser592
30	Gallomyricitrin	−17.11 kcal/mol	Arene-Cation,<br>5H-Bonds	2+Ser592 and 1 H-Bond with Arg583, Asn600, Met601, Ala585
31	Garcinia biflavonoid 2	−12.54 kcal/mol	1AreneCation,<br>2 H-Bonds	Asn600, Arg583, Tyr595
32	Genistein	−12.40 kcal/mol	1 Arene-Cation	Arg583
33	Gossypetin	−14.85 kcal/mol	3H-Bonds	Ser592, Asn600, Ala585
34	Herbacetin	−11.76 kcal/mol	1 H-Bond	Ser592
35	Hidrosmin	−17.51 kcal/mol	3 H-Bonds	Phe594, Asn600, Ala585
36	Hyperoside	−14.44 kcal/mol	3 H-Bonds<br>1 Arene-Cation	Arg583, Thr604, Asn600, Ala585
37	Iscag	−17.63 kcal/mol	2 H-Bonds	Arg583, Met755
38	Isoquercitrin	−15.07 kcal/mol	3 H-Bonds	Asn600, Arg583
39	Isorhamnitin	−13.49 kcal/mol	3 H-Bonds	2 + Thr604 and 1-Asn600
40	Juglanin	−16.63 kcal/mol	4 H-Bonds	2 + Thr604, Arg583, Ser592
41	Kolaflavanone	−11.55 kcal/mol	1 Arene-Cation<br>2 H-Bonds	2 + Arg583, Tyr595
42	Ladanein (BJ486K)	−13.15 kcal/mol	1 Arene-Cation<br>1 H-Bond	Arg583, Thr604
43	Leachianone G	−12.50 kcal/mol	None	-
44	Loquatoside	−14.33 kcal/mol	2 H-Bonds	Asn600, Tyr595
45	Luteolin	−13.33 kcal/mol	1 H-Bond	Thr604
46	Meloside A	−17.92 kcal/mol	4 H-Bonds	2+Asn600, Tyr595, Met755
47	Methyl 6-chloro-3-methoxyflavone-4'-carboxylate	−12.89 kcal/mol	None	-
48	Morin	−13.75 kcal/mol	1 Arene-Cation	Arg583,Thr604,Asn600,Ser592

			3 H-Bonds	
49	Myrciatrin V	−16.39 kcal/mol	3 H-Bonds	2+Asn600,Met601
50				
51	Myricitrin	−16.23 kcal/mol	1 Arene-Cation<br>6 H-Bonds	2+Arg583,Ala584,Ser592,Met601,<br>Thr604,Asn600
52	Narcissin	−16.04 kcal/mol	3 H-Bonds	Arg583,Asn600,Thr604
53	Naringenin	−12.07 kcal/mol	1 H-Bond	Arg583
54	Pachypodol	−12.51 kcal/mol	1 Arene-Cation<br>2 H-Bonds	Arg583,Ala585,Asn600
55	Pectolinarin	−13.55 kcal/mol	3 H-Bonds	Arg583,Ser592,Asn600
56	Quercetagetin	−14.16 kcal/mol	1 Arene-Cation<br>3 H-Bonds	Arg583,Ser592,Asn600,Ala585
57	Quercetin<br>3-(6''-(*E*)-*p*-coumaroylsophoroside)-7-<br>rhamnoside	−18.02 kcal/mol	5 H-Bonds	Ala585, Met601, Thr604,<br>Arg583,Gly584
58	Quercetin-3 β-*O*-D-glucoside	−15.53 kcal/mol	4 H-Bonds	Arg583,Ala585,Asn600,Ala585
59	Quercetin-3,4′-di-*O*-glucoside	−14.74 kcal/mol	5 H-Bonds	2+Asn600.Arg583,Ser592,Tyr595
60	Quercetin	−13.58 kcal/mol	1 H-Bond	Thr604
61	Quercitrin	−14.35 kcal/mol	1 Arene-Cation<br>3 H-Bonds	2+Arg583,2+ Asn600
62	Rhoifolin	−13.21 kcal/mol	2 H-Bonds	Arg583,Ser592
63	Robustaflavone	−19.10 kcal/mol	1 H-Bond	Met755
64	Rutin	−16.53 kcal/mol	1 Arene-Cation<br>4 H-Bonds	2+Thr604, Met601, Arg583<br>Ser592
65	Saponarin	−15.03 kcal/mol	4 H-Bonds	Met755, Asn600, Arg583,<br>Ser592
66	Scutellarein	−14.31 kcal/mol	2 H-Bonds	Met755,Asn600
67	Sigmoidin	−13.63 kcal/mol	3 H-Bonds	2+Arg583,Thr604
68	Silibinin B dihemisuccinate disodium	−12.50 kcal/mol	2 Arene-Cation<br>3 H-Bonds	2+Arg583, Thr604Met601,Ser592
69	Silibinin B dihemisuccinate	−14.95 kcal/mol	1 Arene-Cation<br>4 H-Bonds	2+Arg583, Ser592, Asn600,<br>Gln932
70	Silymarin	−14.66 kcal/mol	1 Arene-Cation<br>3 H-Bonds	Arg583, Ser592, Thr604, Tyr595
71	SRS	−11.67 kcal/mol	4 H-Bonds	Ser592, Asn600, Gly584, Arg583
72	Sulfated Silibinin	−11.66 kcal/mol	2 H-Bonds	Thr604, Ala585
73	PubChem CID-5321560	−11.87 kcal/mol	1 Arene Cation,<br>1 H-Bond	Arg583,Ala585
74	Taiwanhomoflavone A	−13.55 kcal/mol	1 Arene Cation,<br>1 H-Bond	Asn600,Arg583
75	Tetra-*O*-(beta-hydroxyethyl)rutoside	−13.38 kcal/mol	1 Arene Cation,<br>5 H-Bond	3+Arg583,2+Asn600,Thr604
76	Theaflavin 3'-gallate	−16.85 kcal/mol	None	-
77	Theaflavin	−14.04 kcal/mol	1 Arene Cation,<br>2 H-Bond	Arg583,Tyr595,Asn600
78	Tricetin	−10.45 kcal/mol	3 H-Bonds	2+Asn600,Arg583

79	Viscumneoside V	−14.47 kcal/mol	7 H-Bonds	Arg583,Gln932,Gly584,2+Ala585, Tyr595,Ser592
80	Wubangziside C	−15.42 kcal/mol	1 Arene Cation, 5 H-Bonds	3+ Arg583, Ser592, Tyr595,Thr604
81	Zivulgarin	−18.41 kcal/mol	3 H-Bonds	Phe595,Asn600,Met755
82	Dihydromyricetin	−15.32 kcal/mol	1 Arene Cation, 4 H-Bonds	Arg583,Ser592,Met601,Ala585,As n600
83	Hesperetin	−11.96 kcal/mol	1 Arene Cation, 1 H-Bond	Arg583,Thr604
84	Kaempferol	−12.17 kcal/mol	1 H-Bond	Thr604
85	Pinocembrin	−11.49 kcal/mol	1 H-Bond	Arg583
86	Quercetin 7-rhamnoside	−15.92 kcal/mol	5 H-Bonds	Met601,Thr604,Asn600Ala585Phe 594
87	(-)-3,3,4,5,7-Flavanpentol	−11.67 kcal/mol	1 H-bond 1 Arene-Cation	Gly597,Arg583
88	3,7-Dihydroxyflavone	−10.56 kcal/mol	1 Arene-Cation 1 H-bonds	Arg583,Asn600
89	(-)-Epicatechin	−11.78 kcal/mol	1 H-Bond	Arg583
90	(-)-Epigallocatechin gallate (EGCG)	−-14.16 kcal/mol	2 H-bonds	Ser595,Arg583
91	(-)-Epigallocatechin (EGC)	−13.41 kcal/mol	None	-
92	(-)-Gallocatechin gallate (GCG)	−16.14 kcal/mol	3 H-Bonds	Tyr595,Ser592,Thr604
93	3,3',4',5,8-Pentahydroxyflavone	−15.17 kcal/mol	1 Arene-Cation 2 H-bonds	Ser592,Arg583,Thr604
94	3-*O*-methylQuercetin	−15.56 kcal/mol	1 Arene-Cation 3 H-bonds	Ser592, Met601, Arg583, Thr604
95	6-chloro-3-hydroxyflavone-4'-carboxylic acid	−15.93 kcal/mol	1 Arene-Cation 3 H-bonds	Arg583, Thr604, Gly584, Met755
96	7-*O*-(6-Feruoylglucosyl)isoorientin	−18.78 kcal/mol	4 H-Bonds	2 with Asn600,Arg583,Met601
97	Baicalin	−17.79 kcal/mol	5 H-bonds	4+ Asn600 and Ser592,Phe594
98	Cyanidin-3-(*p*-coumaroyl)-rutinoside-5-glucoside	−18.88 kcal/mol	5 H-Bonds	2 with Asn600,Ala581,Arg583,Met755

1

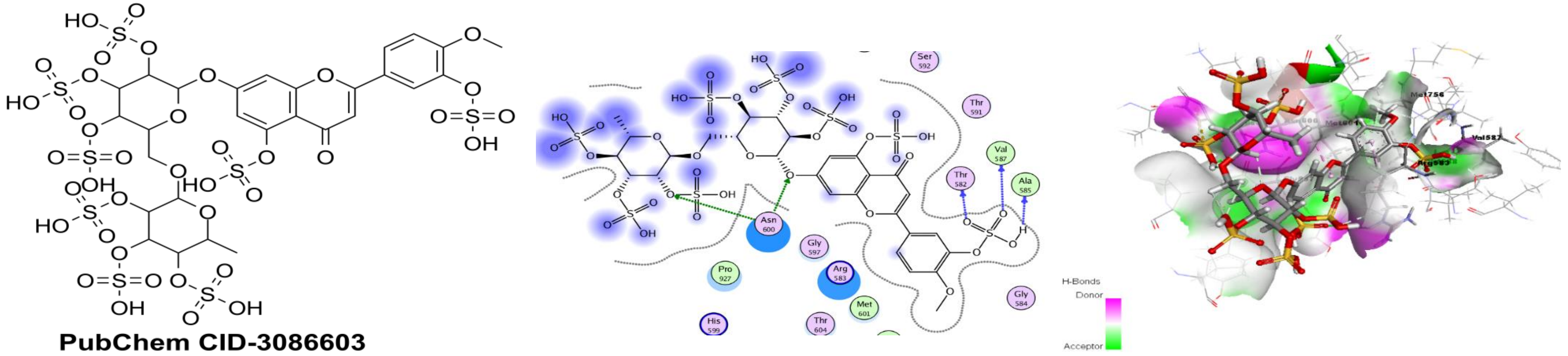
PubChem CID-3086603
Asn 600
Arg 583
Gly 597
Pro 927
His 599
Thr 604
Met 601
Ser 592
Thr 591
Val 587
Thr 582
Ala 585
Gly 584
H-Bonds
Donor
Acceptor

2

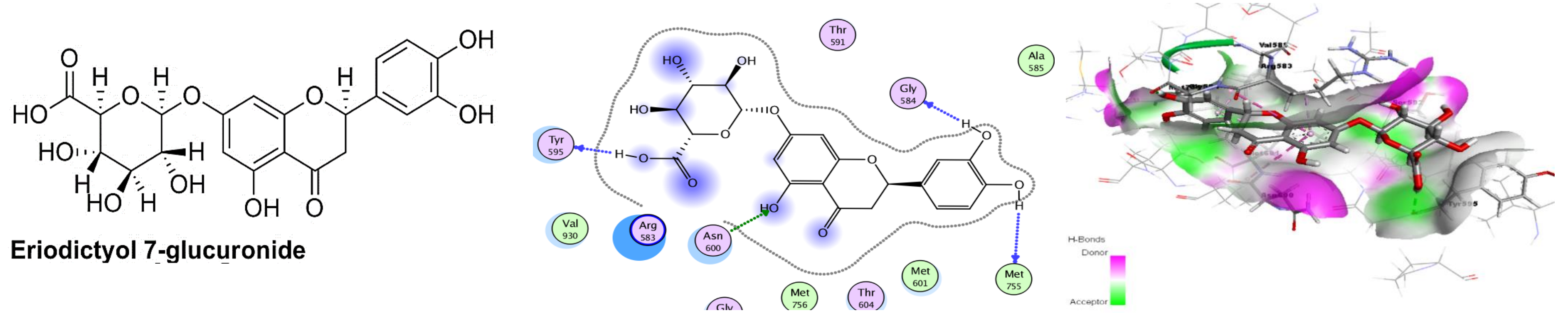
Eriodictyol 7-glucuronide
Thr 591
Ala 585
Gly 584
Tyr 595
Val 930
Arg 583
Asn 600
Met 756
Thr 604
Met 601
Met 755
H-Bonds
Donor
Acceptor

3

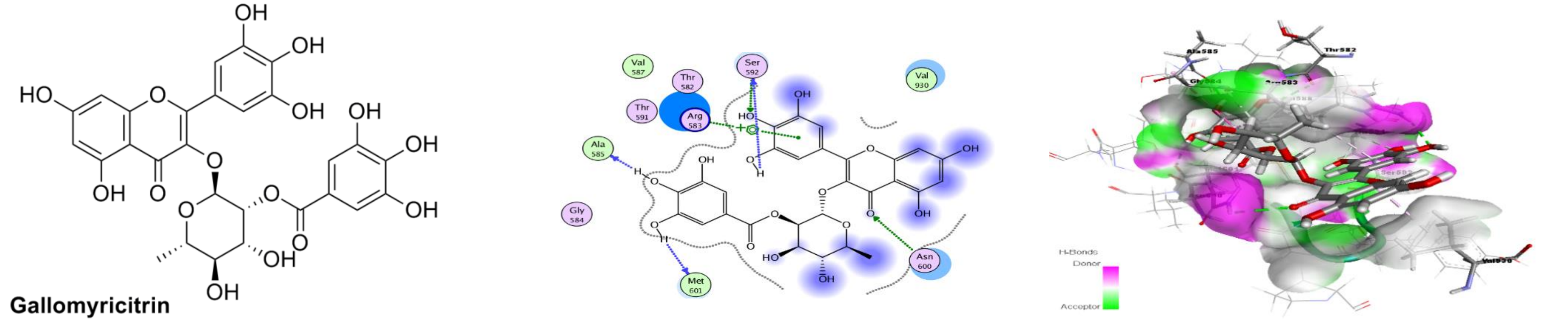
Gallomyricitrin
Val 587
Thr 582
Ser 592
Val 930
Thr 591
Arg 583
Ala 585
Gly 584
Met 601
Asn 600
H-Bonds
Donor
Acceptor

4	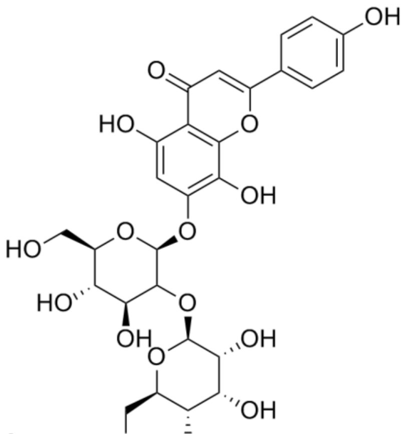 Iscag	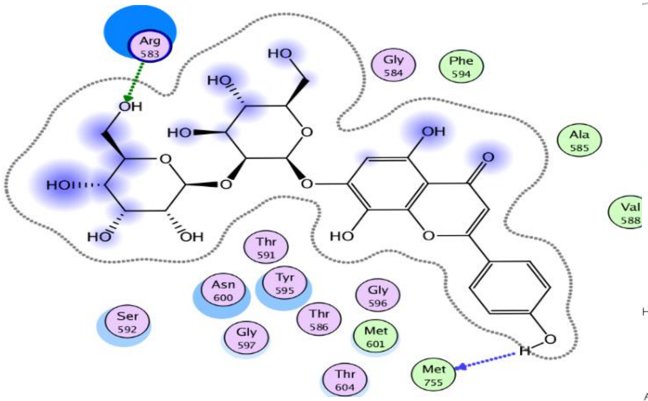	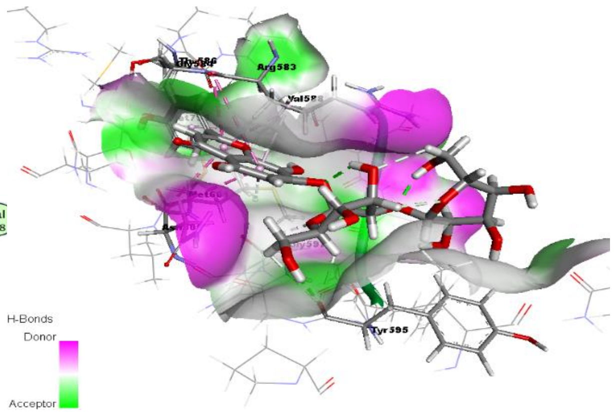
5	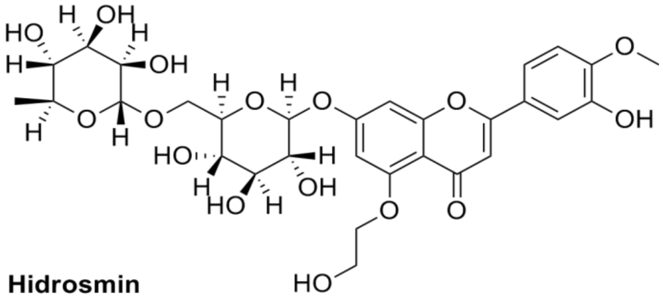 Hidrosmin	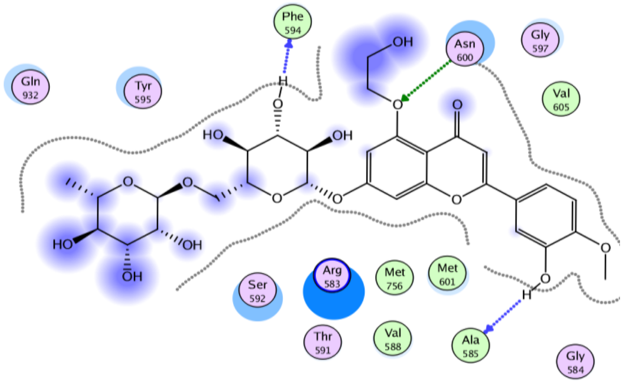	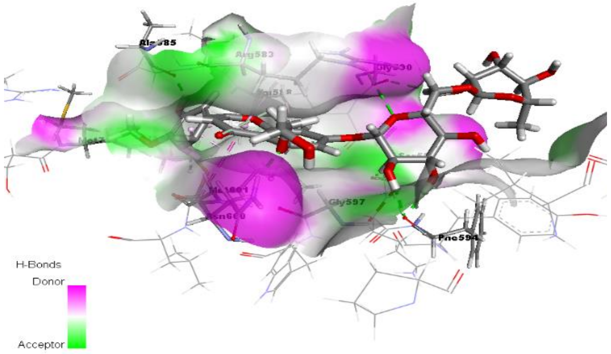

6	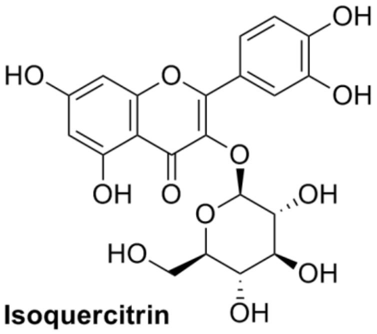 **Isoquercitrin**	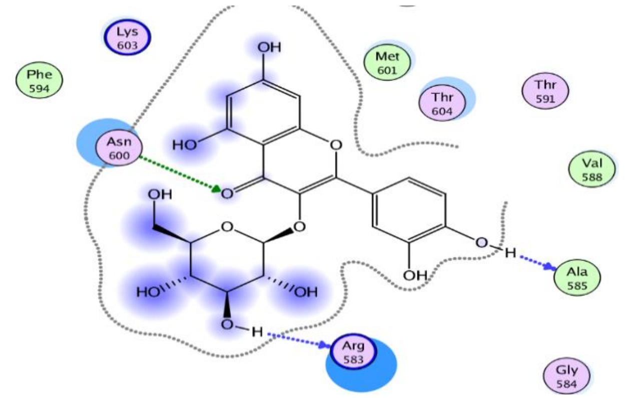	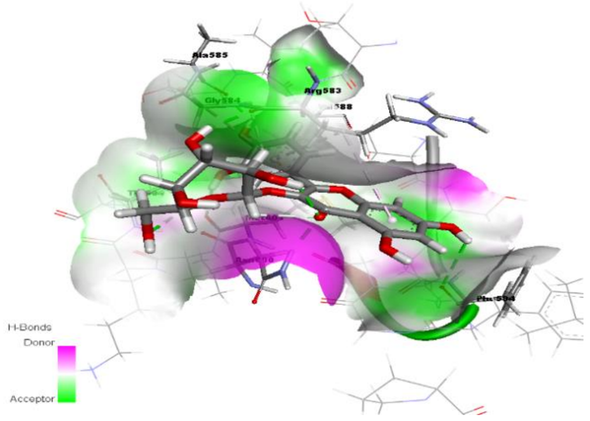
7	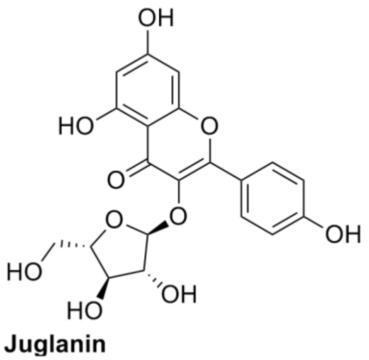 **Juglanin**	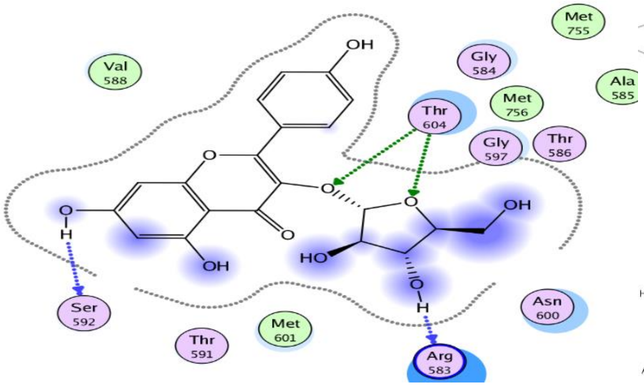	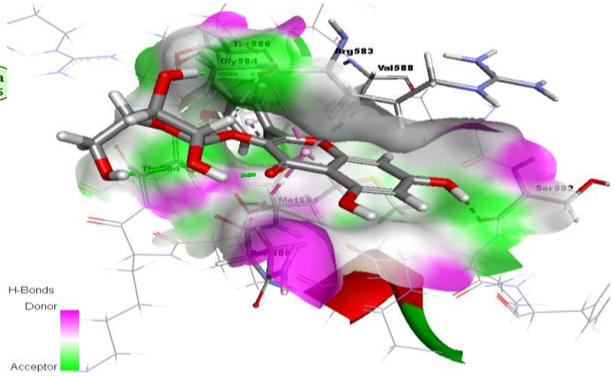

8 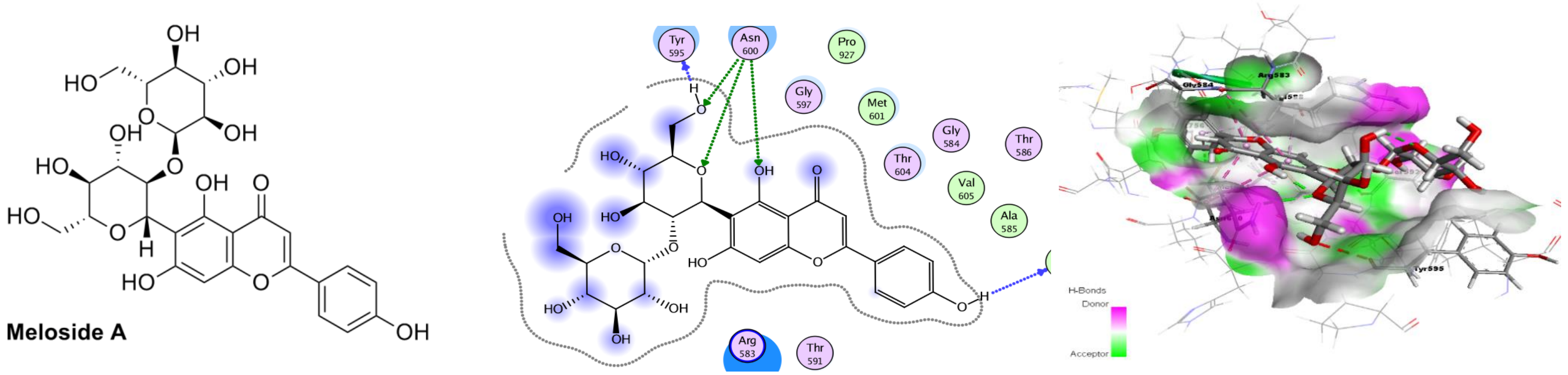
Meloside A
Tyr 595
Asn 600
Pro 927
Gly 597
Met 601
Gly 584
Thr 586
Thr 604
Val 605
Ala 585
Arg 583
Thr 591
H-Bonds
Donor
Acceptor

9 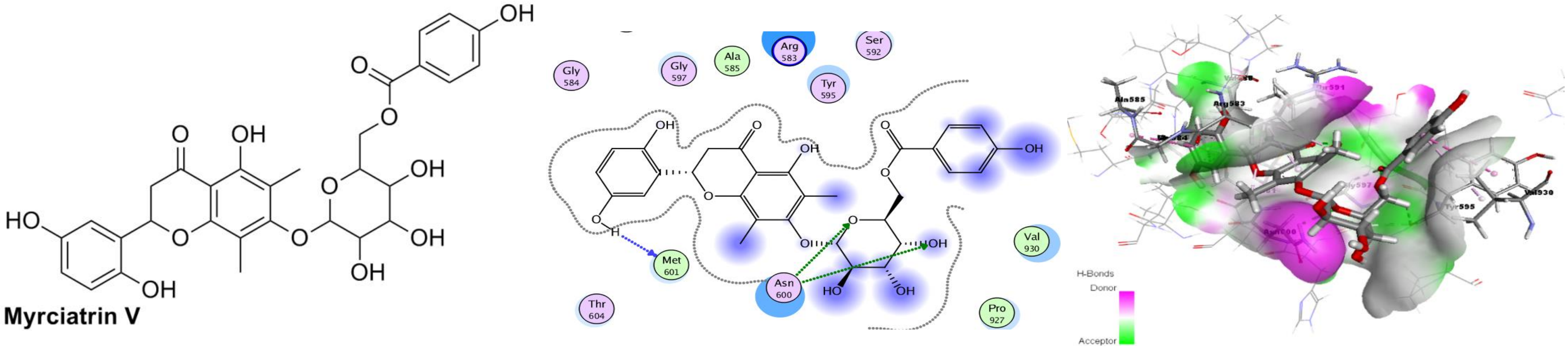
Myrciatrin V
Gly 584
Gly 597
Ala 585
Arg 583
Ser 592
Tyr 595
Met 601
Asn 600
Val 930
Thr 604
Pro 927
H-Bonds
Donor
Acceptor

10

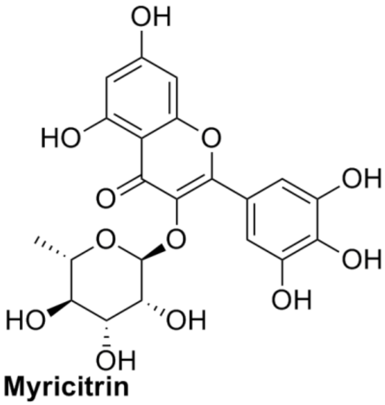
Myricitrin

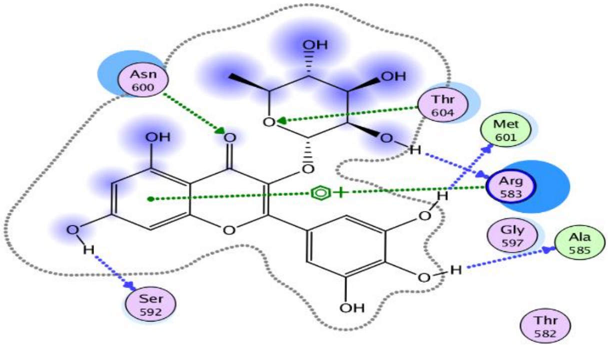
Asn 600
Thr 604
Met 601
Arg 583
Gly 597
Ala 585
Ser 592
Thr 582

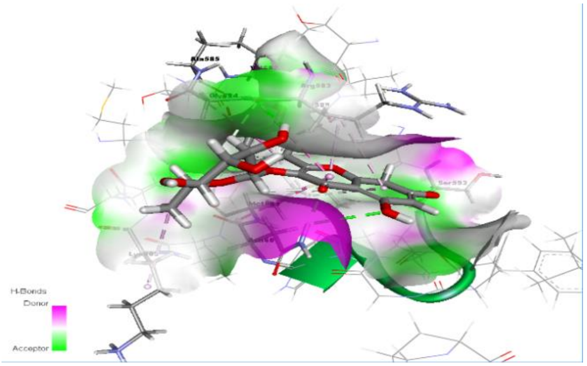
H-Bonds
Donor
Acceptor

11

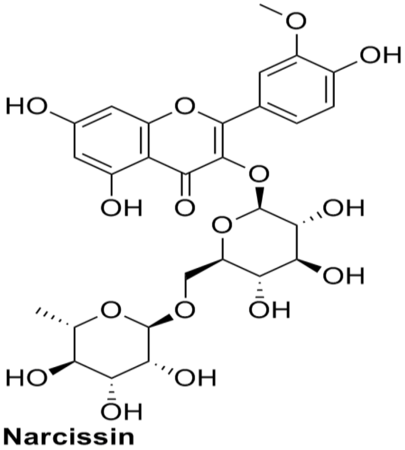
Narcissin

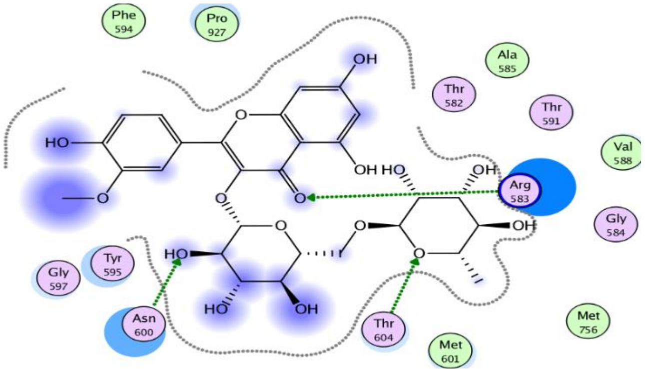
Phe 594
Pro 927
Ala 585
Thr 582
Thr 591
Val 588
Arg 583
Gly 584
Gly 597
Tyr 595
Asn 600
Thr 604
Met 601
Met 756

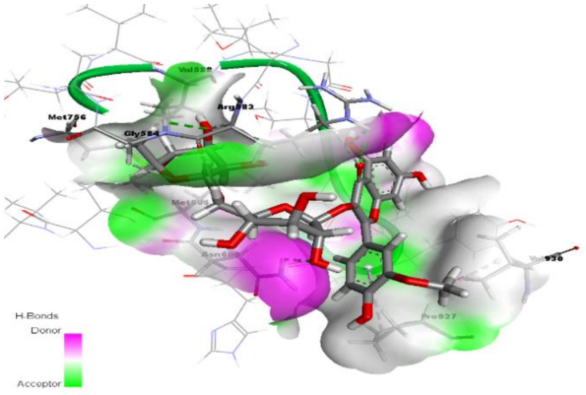
H-Bonds
Donor
Acceptor

12 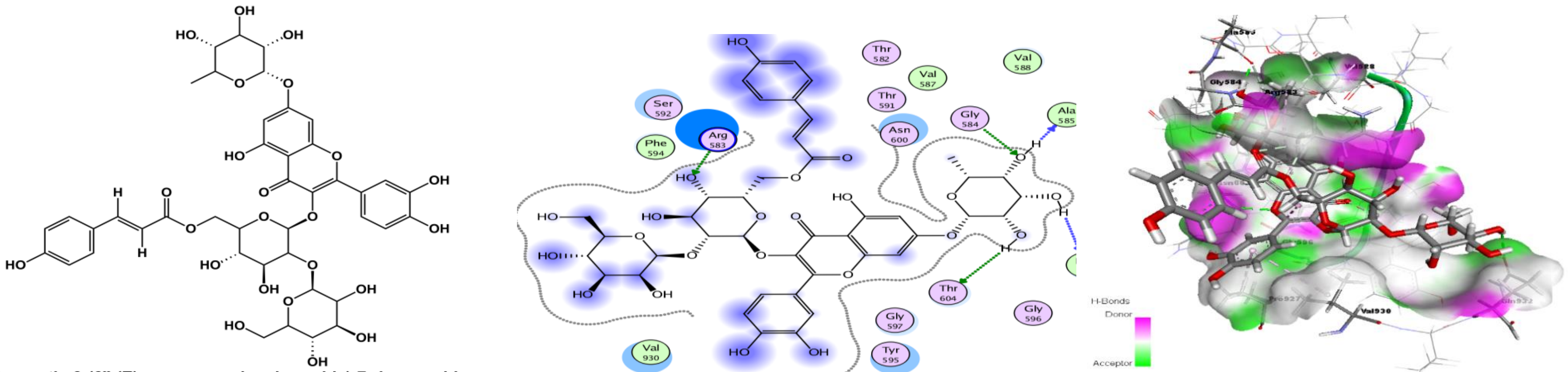

Quercetin 3-(6''-(E)-p-coumaroylsophoroside)-7-rhamnoside

13 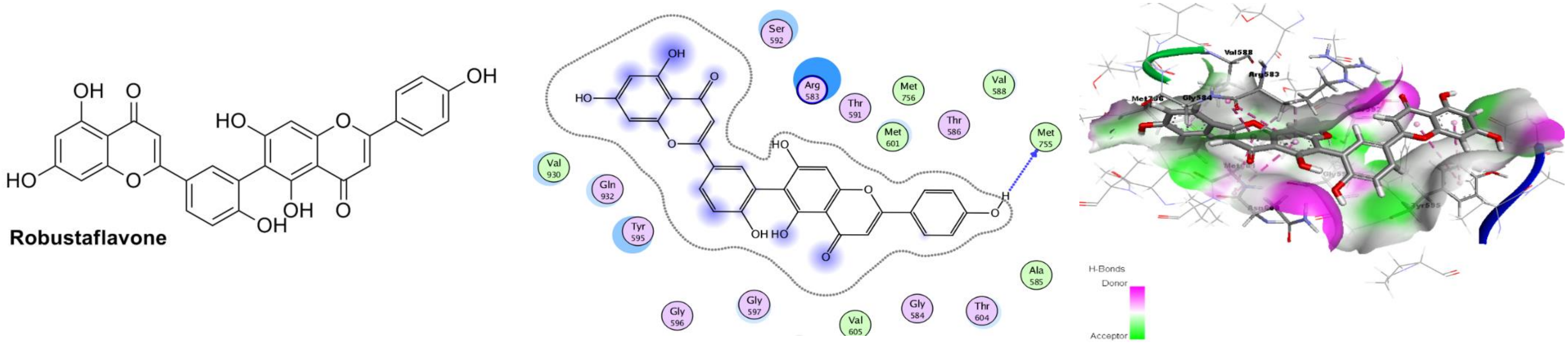

14	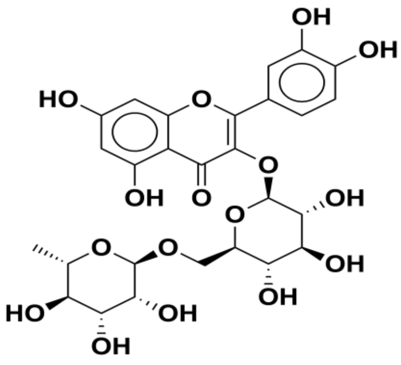 Rutin	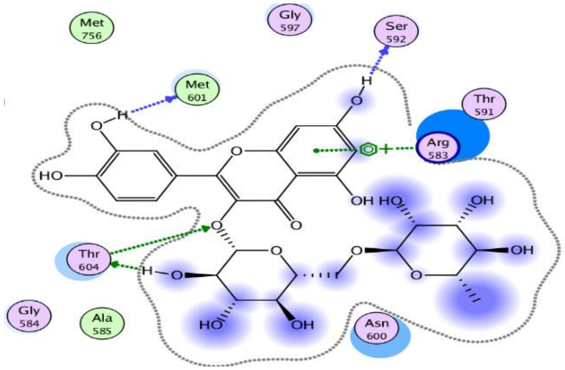	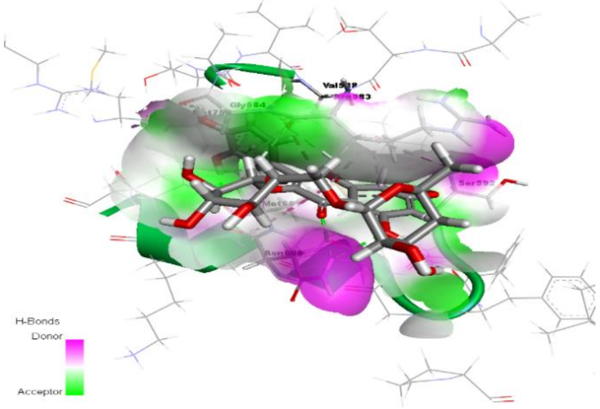
15	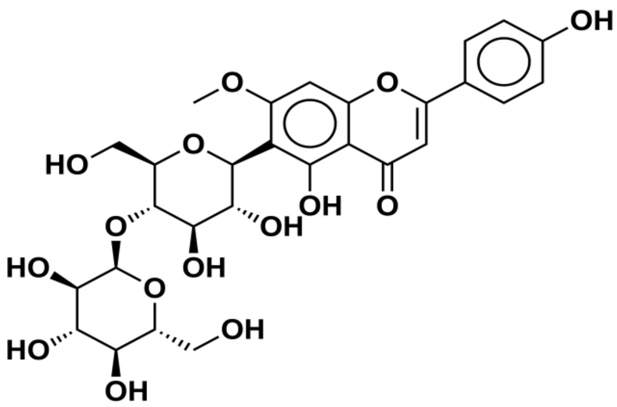 Zivulgarin	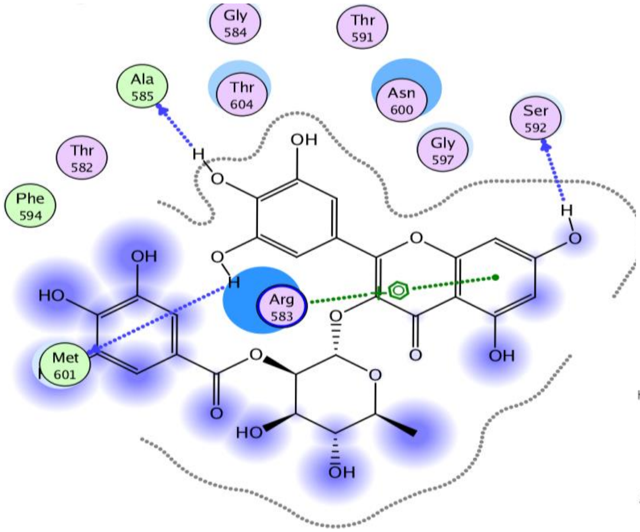	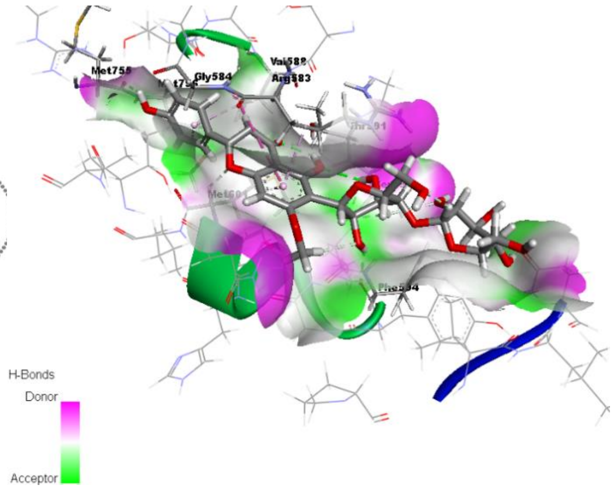

16

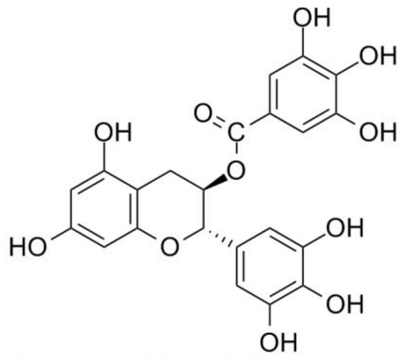

Gallocatechin gallate.

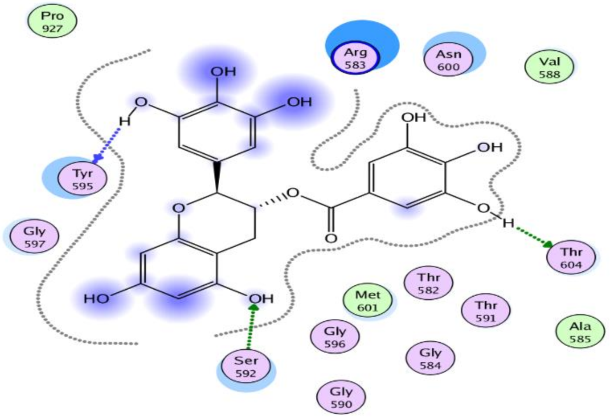

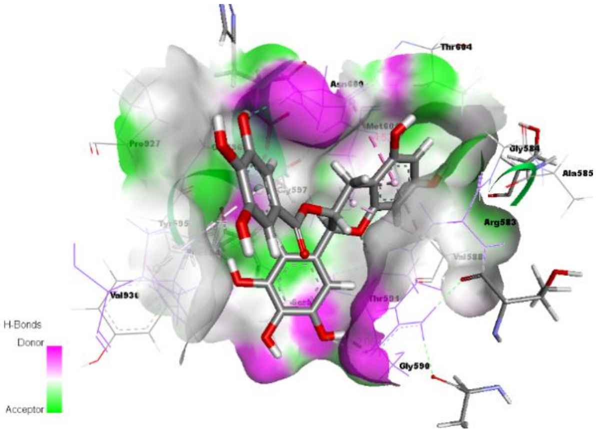

17

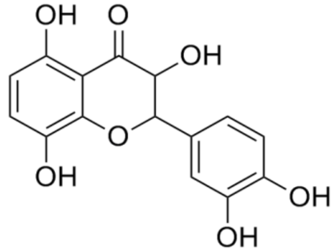

3,3',4',5,8-Pentahydroxyflavone

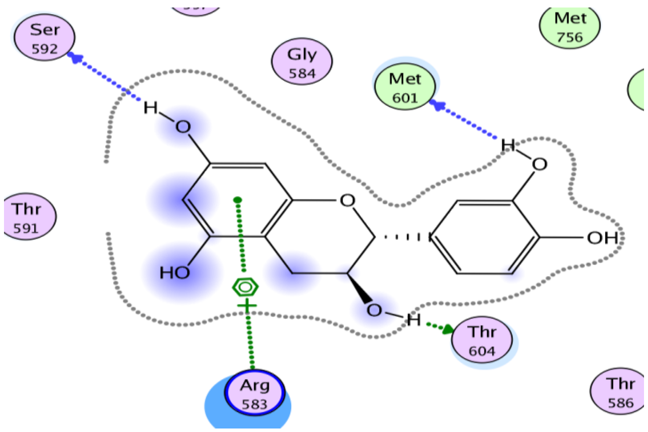

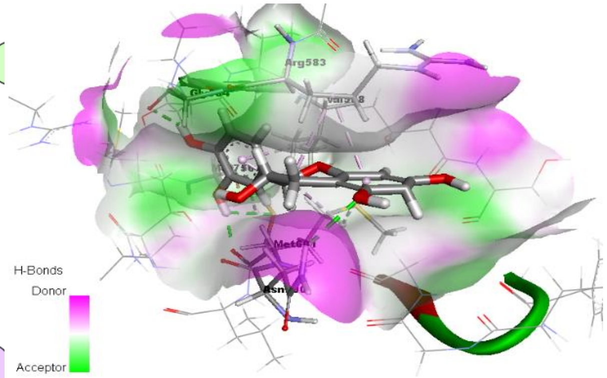

18

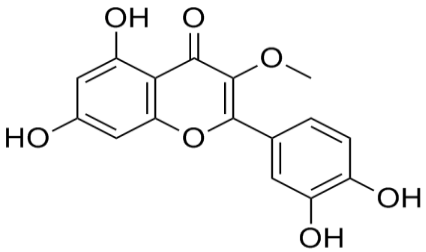

3-O--methylQuercetin

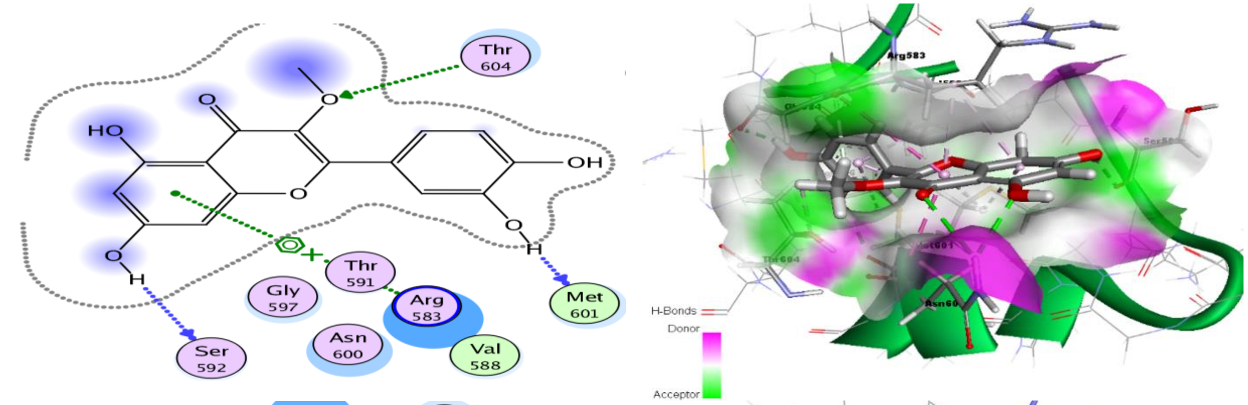

19

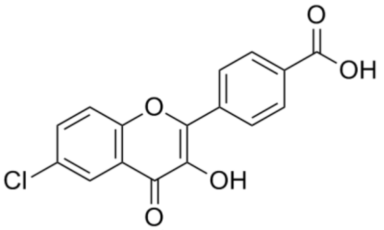

6-chloro-3-hydroxyflavone-4'- carboxylic acid

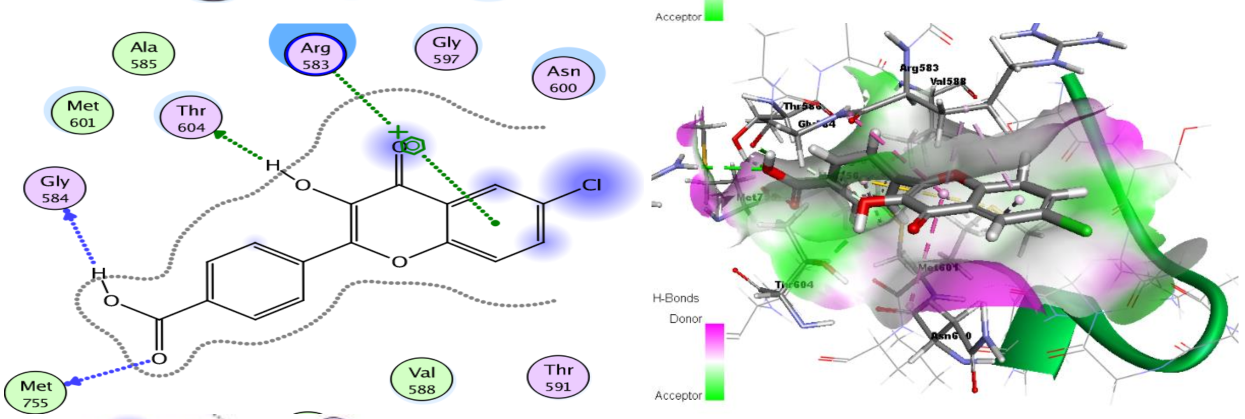

20

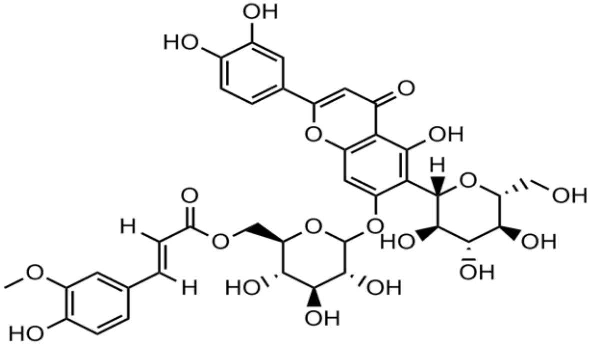

7-O-(6-Feruoylglucosyl)isoorientin

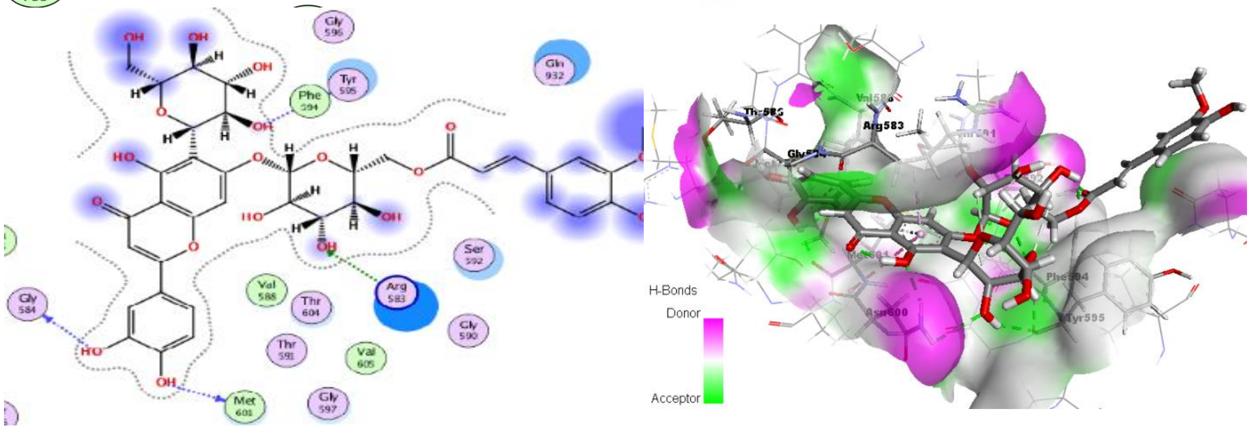

21 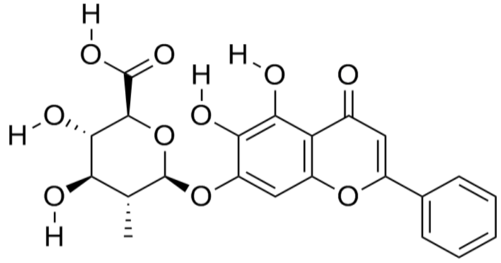

Baicalin

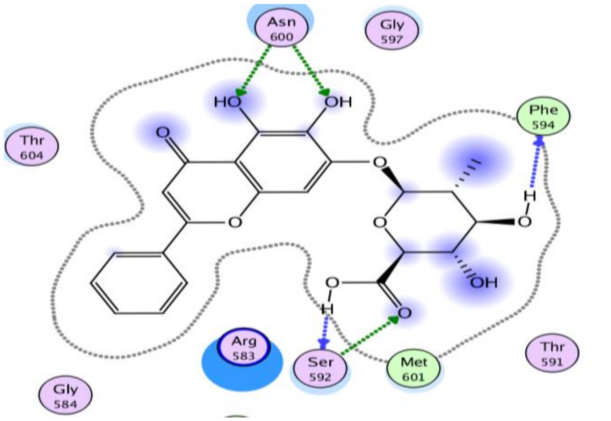

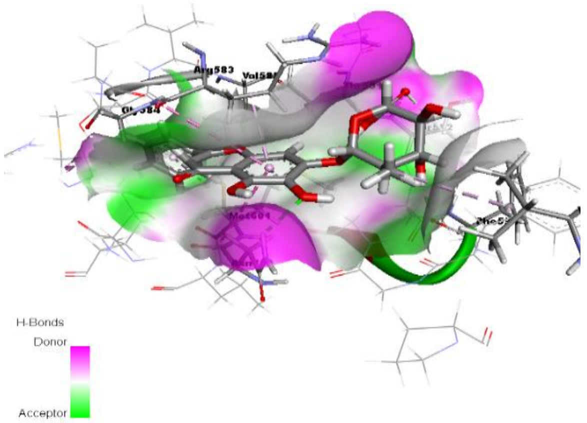

22 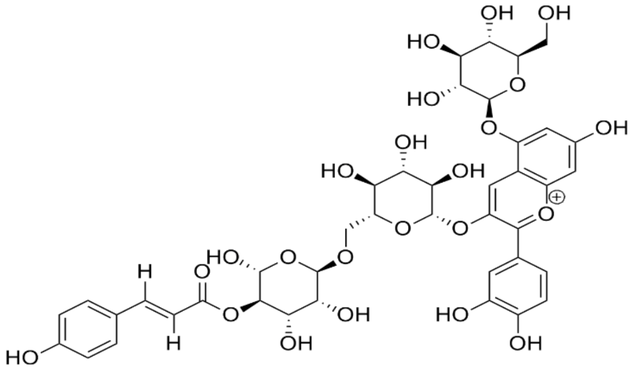

Cyanidin-3-(p-coumaroyl)-rutinoside-5-glucoside

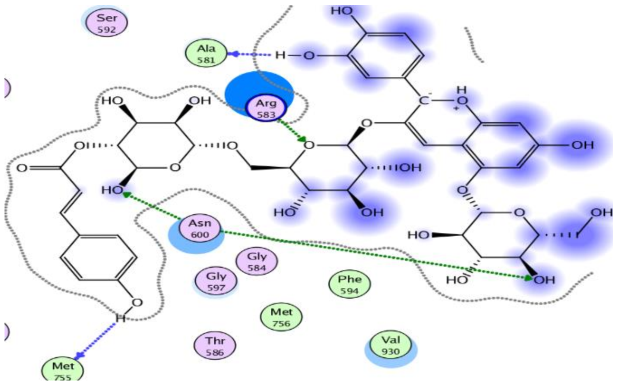

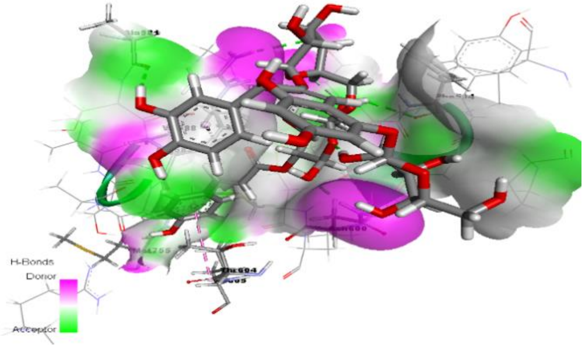

Figure S1. The docked flavonoid molecules in 2D and 3D structure inside the allosteric binding pocket.

molecules

MDPI

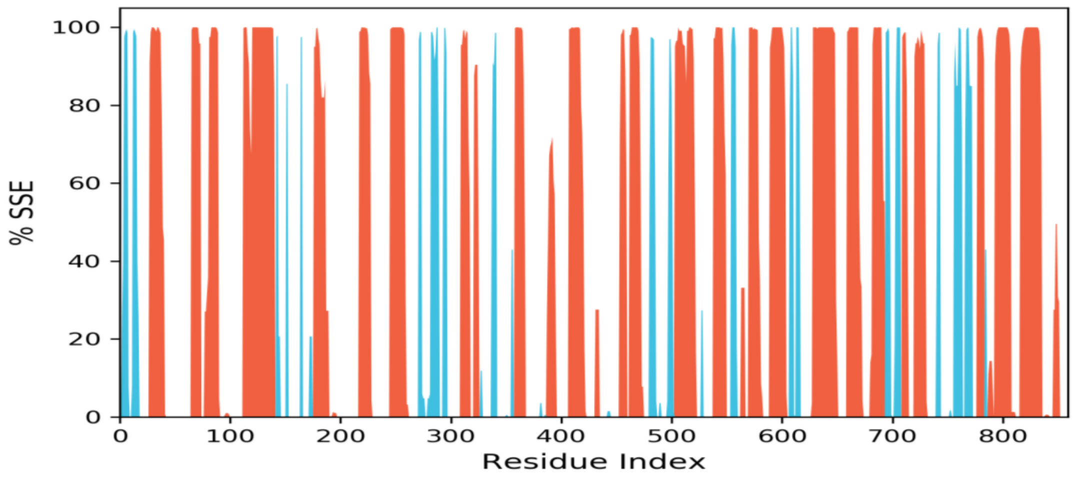

Figure S2. Percentage of the secondary structure elements of the residue of the apo-enzyme.

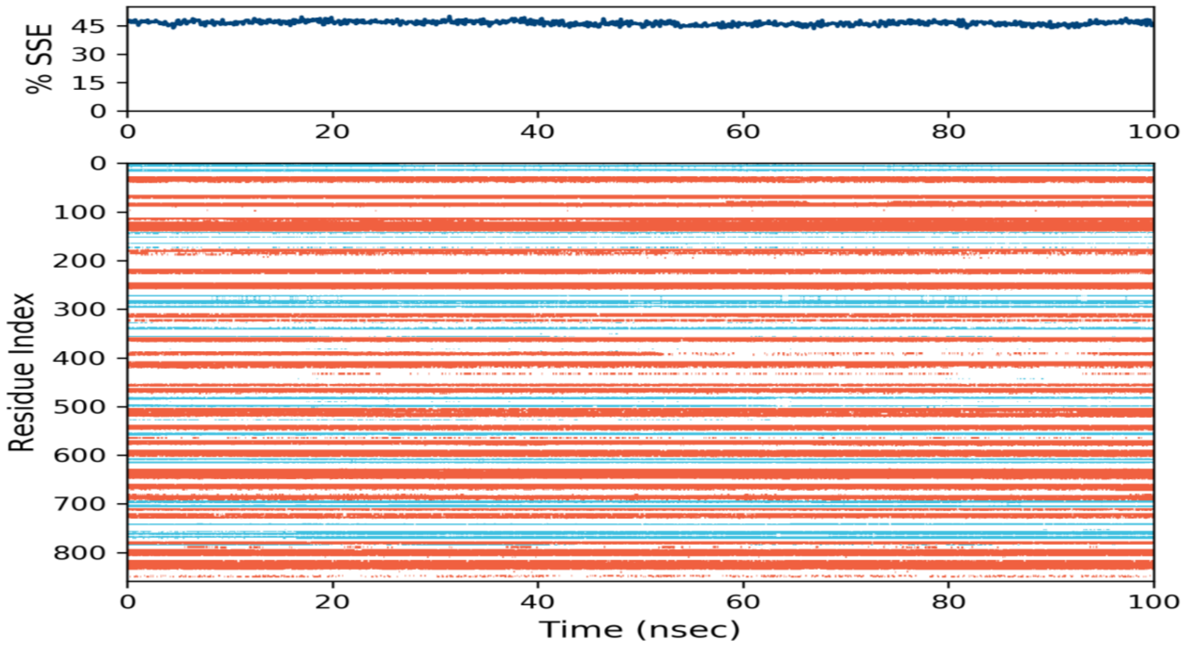

Figure S3. Secondary structure elements residue index of apo-protein as a function of simulation time.

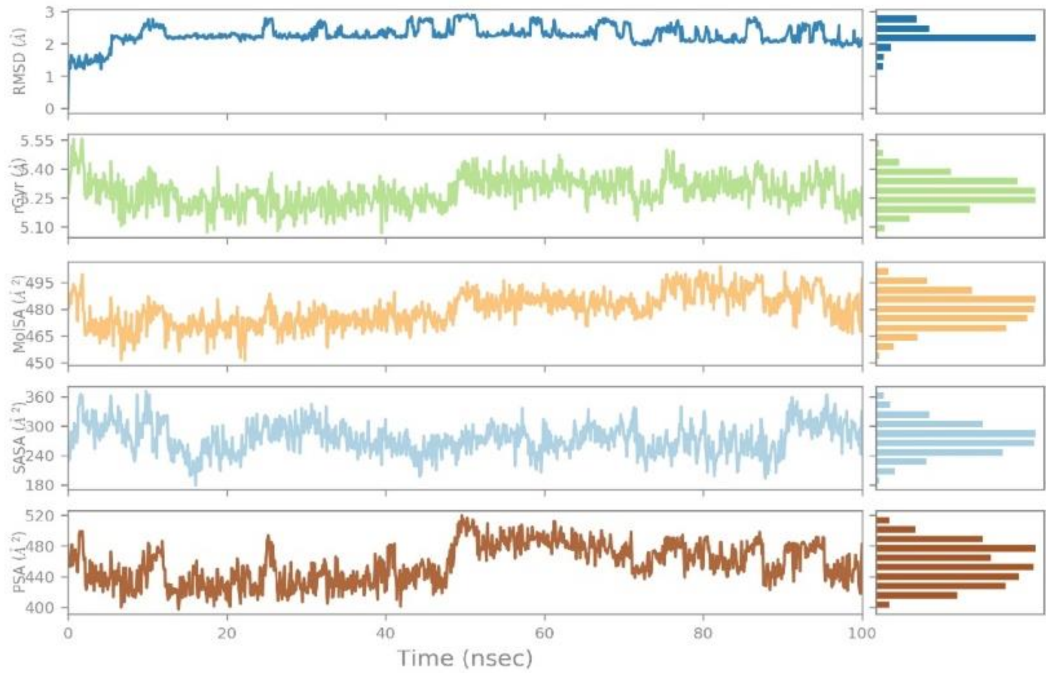

Figure S4. The polar surface area (PSA); solvent accessible surface area (SASA), and the van der Waals surface area (MolSA) of the RdRp in complex with aureusidin 4, 6-diglucoside.

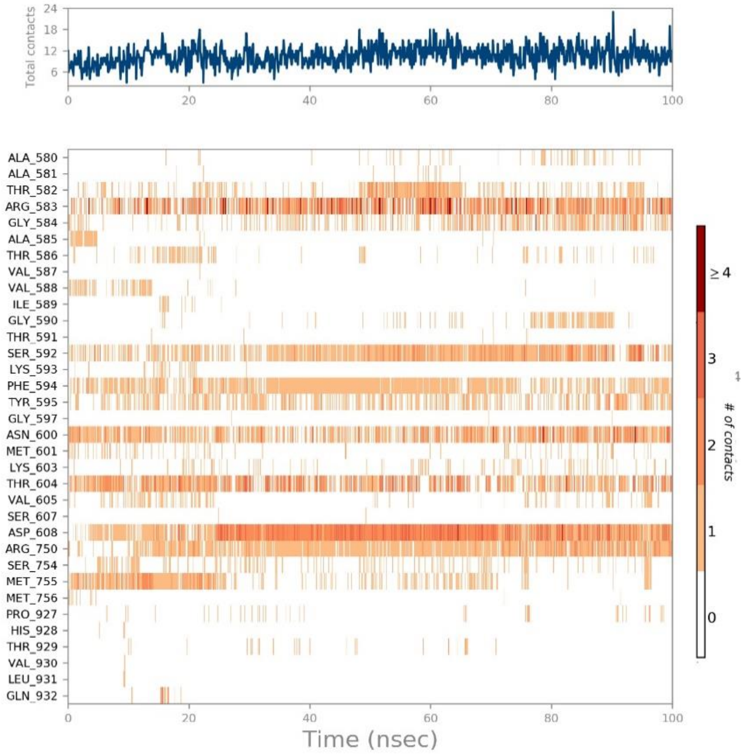

Figure S5. Contacts of the different residue and the ligand during the 100 ns simulation time.

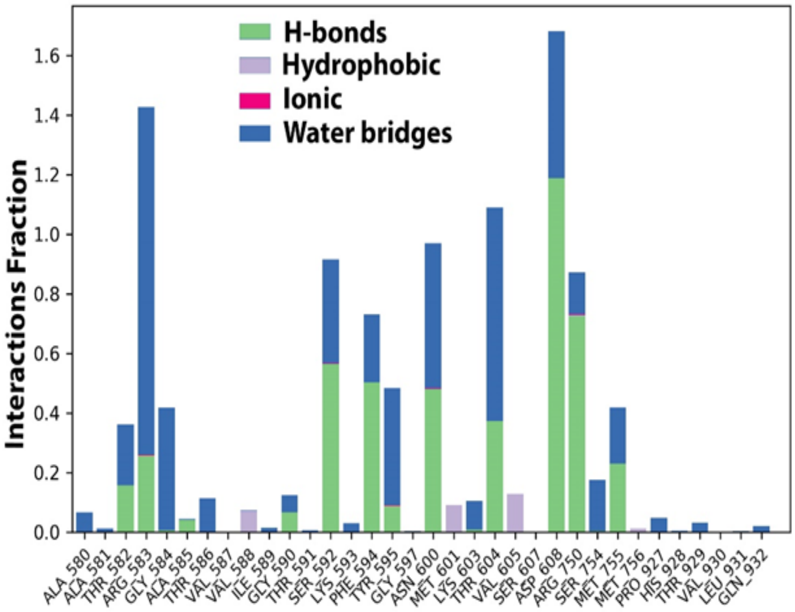

Figure S6. Various types of interaction made by the amino acid residues of the protein during the simulation.

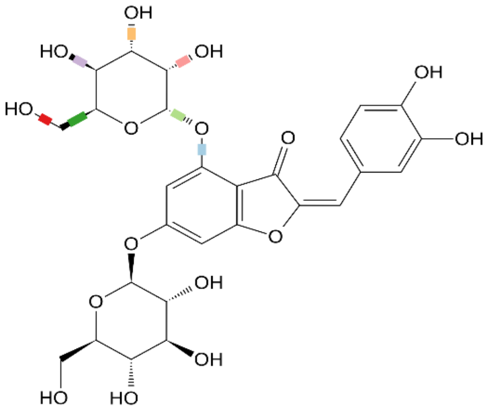

Figure S7. Torsion and flexibility in the aureusidin 4, 6-diglucoside.

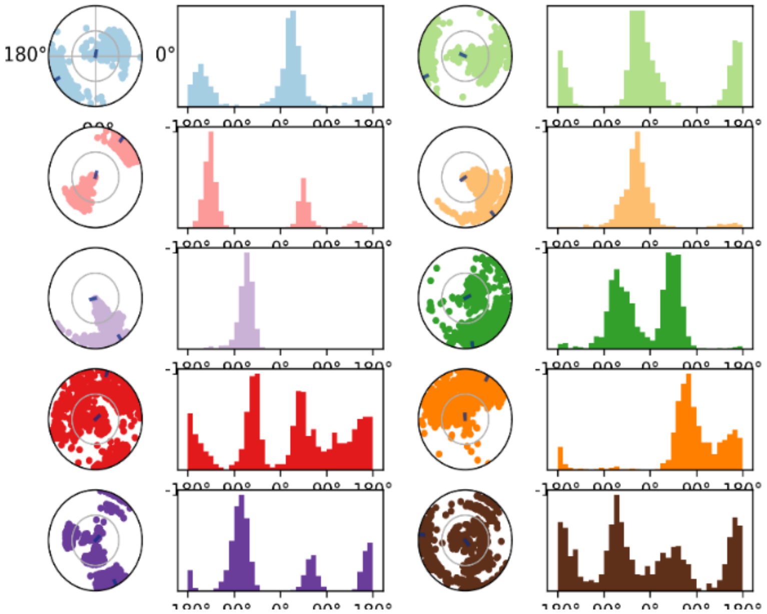

Figure S8. Torsional angles of the aureusidin 4, 6-diglucoside flavonoid.

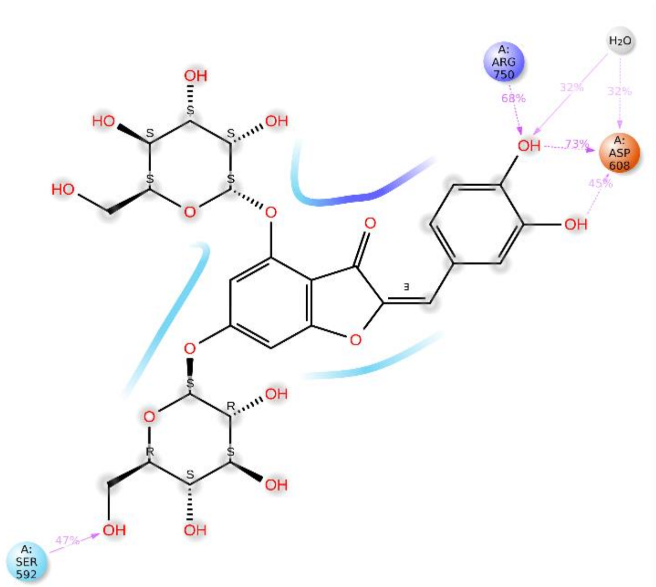

Figure S9. Various types of interaction made by the aureusidin 4, 6-diglucoside with the amino acid residues of the protein during the simulation.

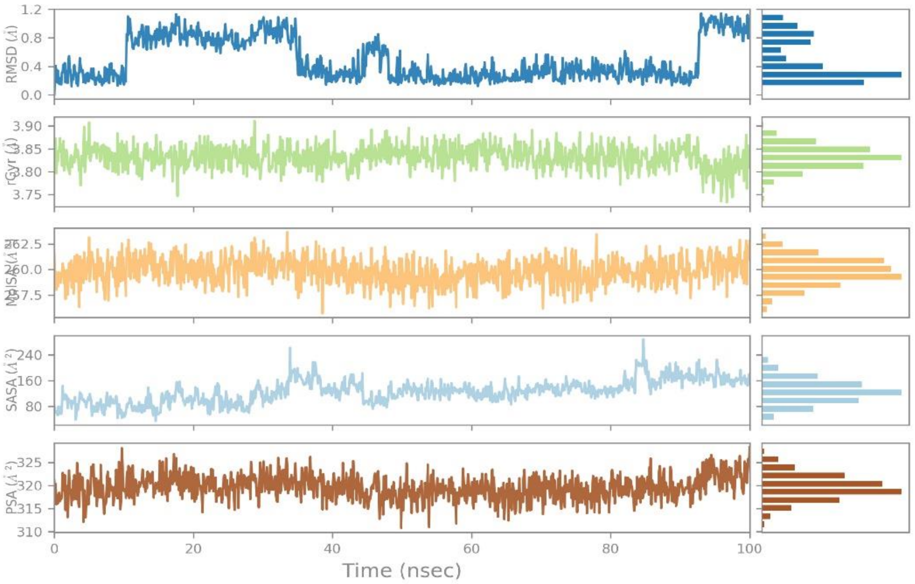

Figure S10. Polar Surface Area (PSA), solvent accessible surface area, the van der Waals surface area, (MolSA), radius of gyration and RMSD of the RdRp in complex with the myricetin during 100 ns simulation.

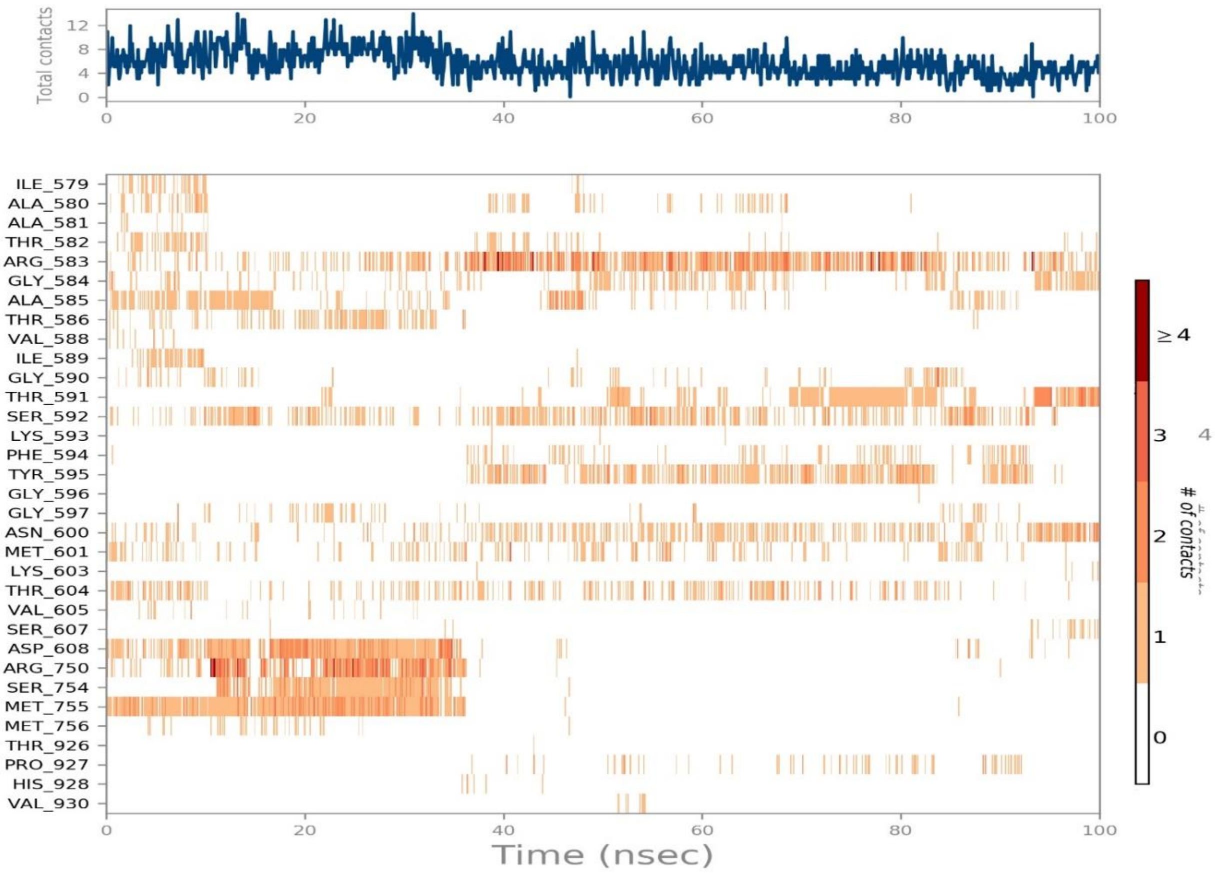

Figure S11. Allosteric residues contacts of the RdRp with the myricetin flavonoid ligand.

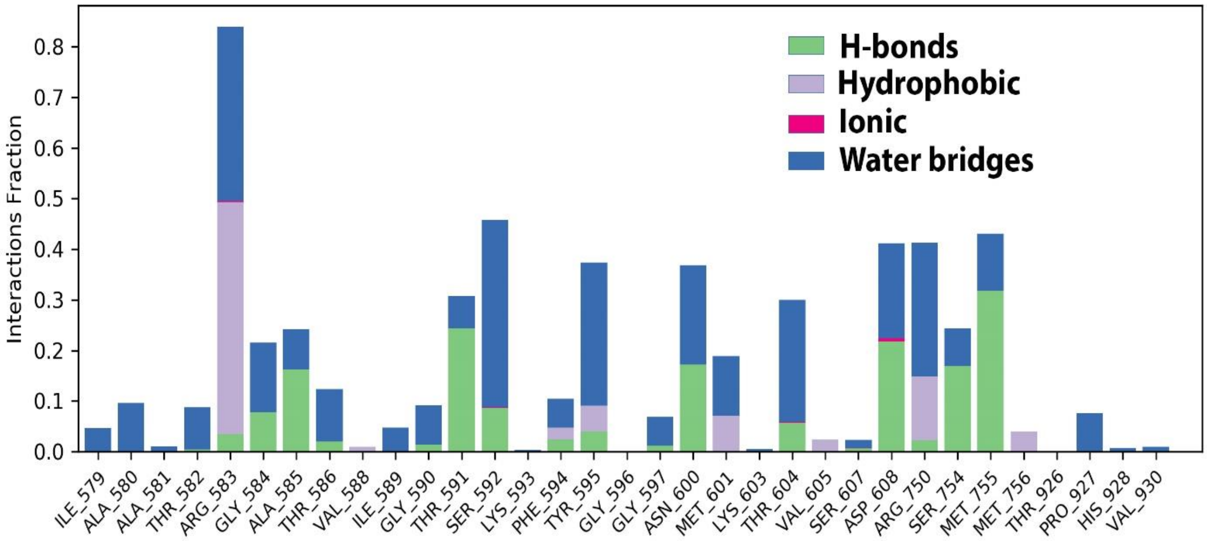

Figure S12. Various types of interactions made by specific amino acids with the myricetin flavonoid.

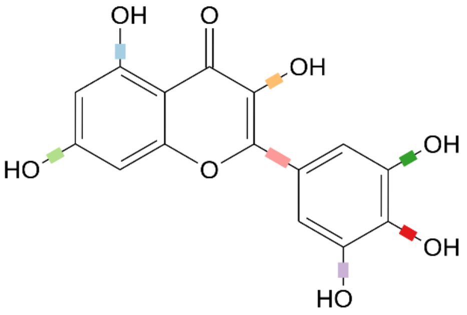

Figure S13. Myricetin torsions that are involved in its fluctuation inside the protein.

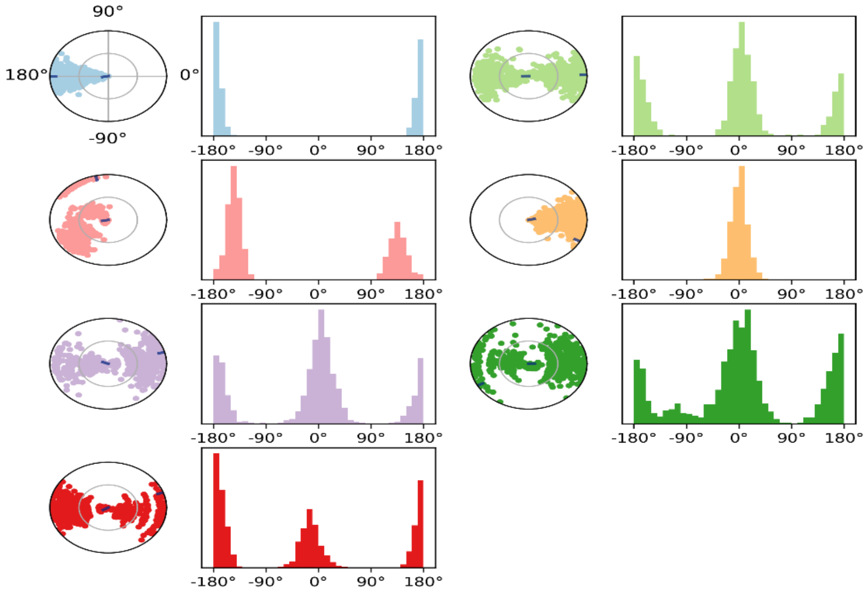

Figure S14. Torsional angles of the myricetin.

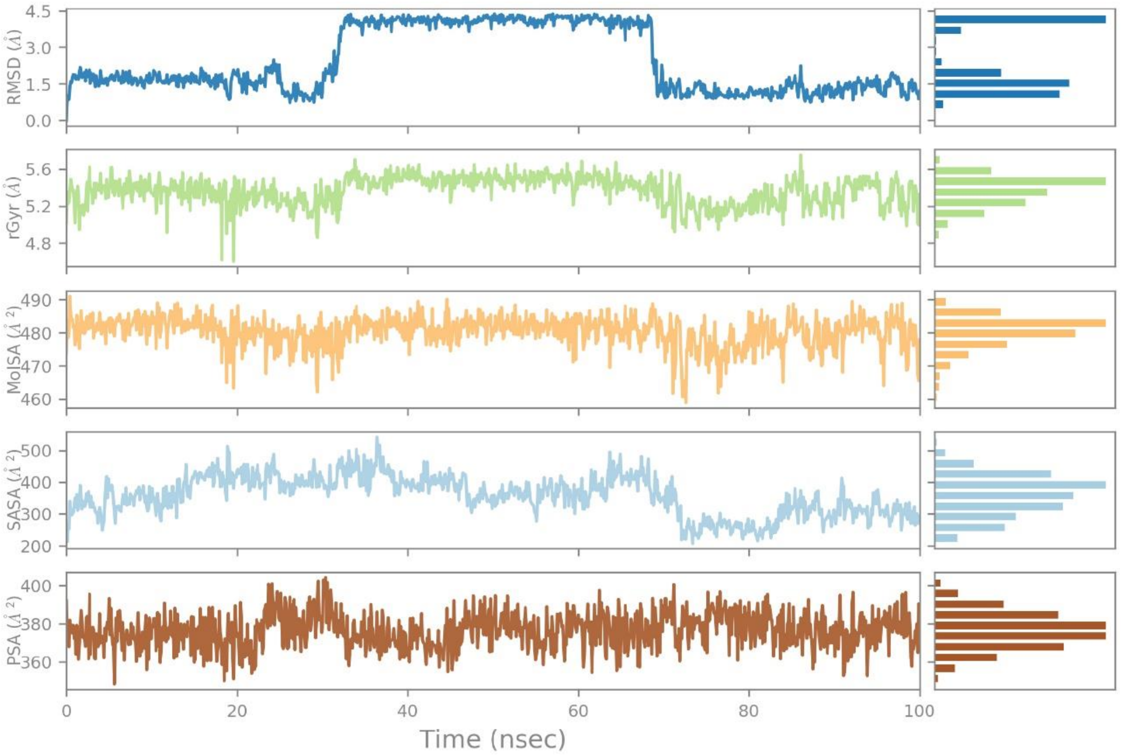

Figure S15. Polar surface area, solvent accessible surface area, the van der Waals surface area, radius of gyration and RMSD of the RdRp in complex with the naringoside during 100 ns simulation.

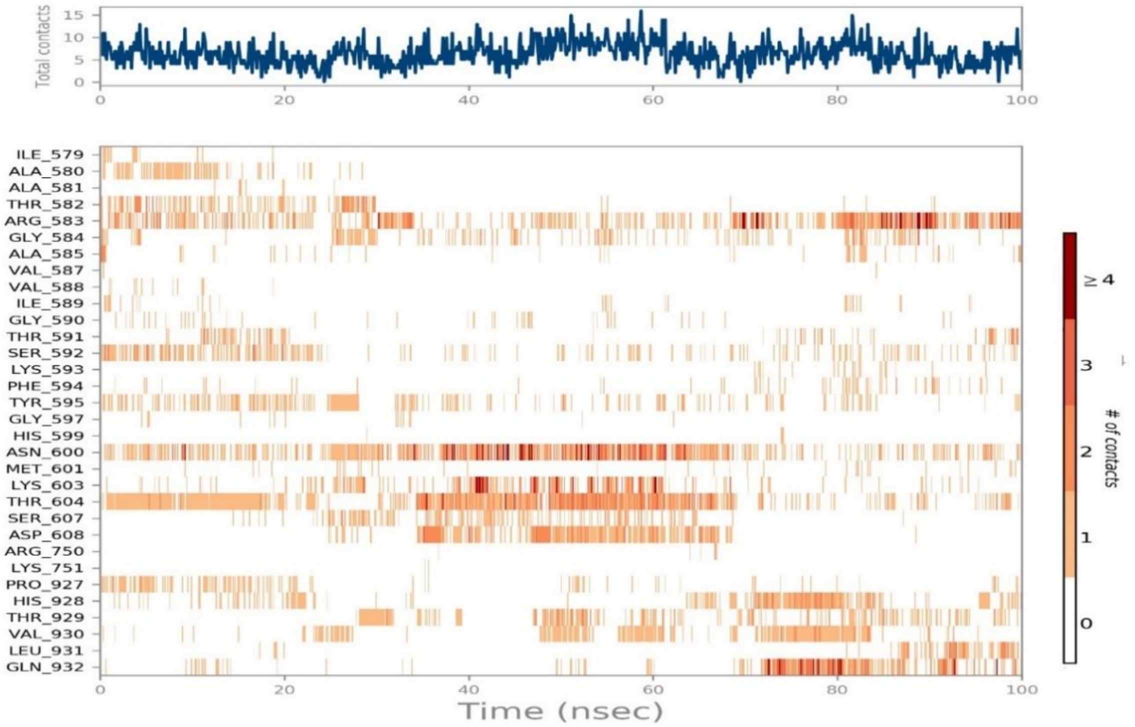

Figure S16. Number of contacts made by the allosteric and other residues of RdRp.

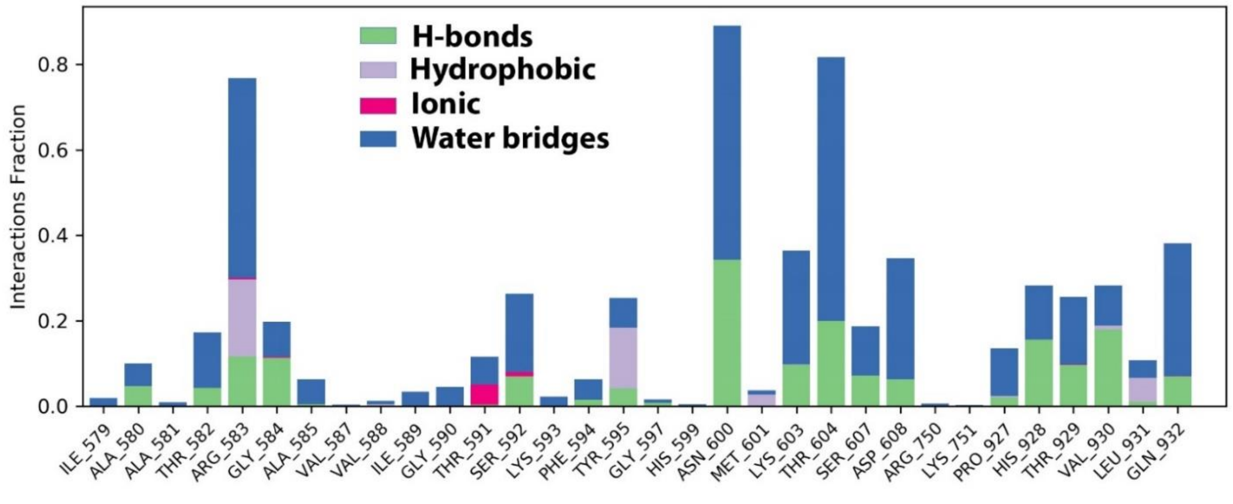

Figure S17. Various types of interactions and its total fraction made by the allosteric residues with ligand.

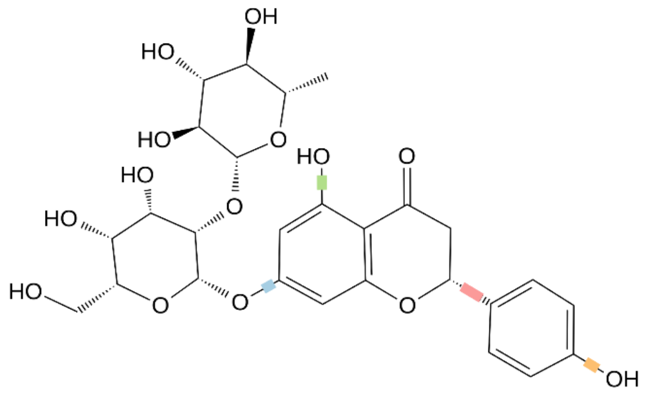

Figure S18. Torsion angles of the naringoside ligand.

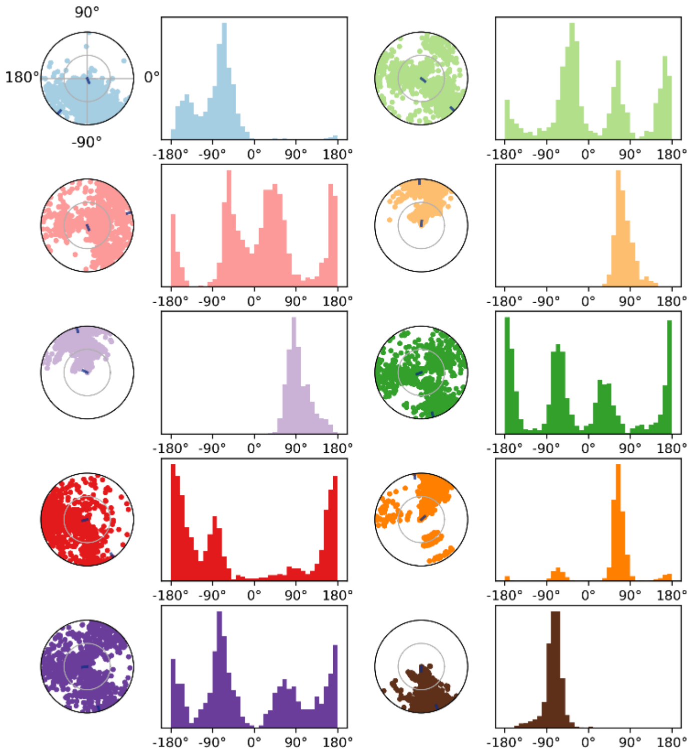

Figure S19. Torsional angles of the narigoside.
